# Testing and Treatment Interventions in Community Settings Key to Controlling a Recent Human Immunodeficiency Virus Outbreak Among People Who Inject Drugs in Glasgow: A Modeling Study

**DOI:** 10.1093/infdis/jiae206

**Published:** 2024-05-22

**Authors:** Lara I Allen, Hannah Fraser, Jack Stone, Andrew McAuley, Kirsten M A Trayner, Rebecca Metcalfe, S Erica Peters, Sharon J Hutchinson, Peter Vickerman, Matthew Hickman

**Affiliations:** Population Health Sciences, Bristol Medical School, University of Bristol; Clinical and Protecting Health Directorate, Public Health Scotland; Population Health Sciences, Bristol Medical School, University of Bristol; Population Health Sciences, Bristol Medical School, University of Bristol; Clinical and Protecting Health Directorate, Public Health Scotland; School of Health and Life Sciences, Glasgow Caledonian University; Clinical and Protecting Health Directorate, Public Health Scotland; School of Health and Life Sciences, Glasgow Caledonian University; School of Health and Life Sciences, Glasgow Caledonian University; Sandyford Sexual Health Service, National Health Service Greater Glasgow and Clyde; Brownlee Centre for Infectious Diseases, National Health Service Greater Glasgow and Clyde, Glasgow, United Kingdom; Clinical and Protecting Health Directorate, Public Health Scotland; School of Health and Life Sciences, Glasgow Caledonian University; Population Health Sciences, Bristol Medical School, University of Bristol; Population Health Sciences, Bristol Medical School, University of Bristol

**Keywords:** HIV, modeling, antiretroviral treatment, HIV testing

## Abstract

**Background:**

A human immunodeficiency virus (HIV) outbreak was identified among people who inject drugs (PWID) in Glasgow in 2015, with >150 diagnoses by the end of 2019. The outbreak response involved scaling up HIV testing and improving HIV treatment initiation and retention.

**Methods:**

We parameterized and calibrated a dynamic, deterministic model of HIV transmission among PWID in Glasgow to epidemiological data. We use this model to evaluate HIV testing and treatment interventions. We present results in terms of relative changes in HIV prevalence, incidence, and cases averted.

**Results:**

If the improvements in both testing and treatment had not occurred, we predict that HIV prevalence would have reached 17.8% (95% credible interval [CrI], 14.1%–22.6%) by the beginning of 2020, compared to 5.9% (95% CrI, 4.7%–7.4%) with the improvements. If the improvements had been made on detection of the outbreak in 2015, we predict that peak incidence would have been 26.2% (95% CrI, 8.8%–49.3%) lower and 62.7% (95% CrI, 43.6%–76.6%) of the outbreak cases could have been averted. The outbreak could have been avoided if the improvements had already been in place.

**Conclusions:**

Our modeling suggests that the HIV testing and treatment interventions successfully brought the HIV outbreak in Glasgow under control by the beginning of 2020.

The potential for human immunodeficiency virus (HIV) transmission via sharing injecting equipment means that people who inject drugs (PWID) are at particular risk of acquiring HIV [1], with an estimated 15% of PWID globally living with the infection [2]. Harm reduction interventions, such as opioid agonist therapy (OAT) and needle and syringe programs (NSPs), can reduce the risk of blood-borne virus (BBV) transmission among this population [3]. OAT effectively reduces transmission by reducing injecting frequency, while NSPs reduce the number of needle/syringe sharing events. Suboptimal harm reduction coverage has contributed to several recent HIV outbreaks among PWID internationally [4].

Despite high levels of harm reduction coverage [4], an HIV outbreak among PWID was identified in the Greater Glasgow and Clyde (GGC) region of Scotland in 2015 [5, 6], leading to an HIV prevalence of more than 10% in Glasgow City Centre (GCC), approximately 10 times pre-outbreak levels [5].

Analysis of the GGC outbreak found associations between HIV and multiple risk factors, including cocaine injecting (which increased rapidly over the course of the outbreak), homelessness, frequent incarceration, and public injecting [5, 7]. Stimulant injecting has been associated with many recent outbreaks of HIV [4] and is linked with higher injecting frequency [8].

Before detection of the outbreak, HIV testing rates among PWID in GCC were low, with approximately 30% tested in the last year prior to the outbreak [9]. To increase HIV testing, key interventions included the systematic expansion of testing in drug treatment services and opt-out BBV testing in prisons. The proportion of PWID reporting a recent HIV test in GCC more than doubled (from 30% to nearly 70%), but was slow to rise during the first years of the outbreak [9]. Testing rates in the Rest of Greater Glasgow and Clyde (RoGGC) also increased but to a lesser extent [9].

Antiretroviral treatment (ART) is a highly effective treatment for HIV. When adherence to treatment is good, the virus will be suppressed and HIV transmission does not occur [10]. Treatment as prevention is effective because it reduces the population-level viral load, which reduces the number of onward transmissions. ART was available for PWID in GGC prior to 2015, but treatment and viral suppression rates were low among the early outbreak cases in 2014–2015 [11]. The original treatment model, which was delivered via hospitals, was found to be insufficient to meet the needs of the population. Therefore, the Glasgow Enhanced Care HIV Outreach (GECHO) treatment model was developed, which involved recruiting a BBV clinical nurse specialist and implementing a consultant-led HIV clinical service in close proximity to the target population. Community pharmacy services were also adapted to enhance ART adherence by providing ART alongside OAT, which was supervised and delivered daily for the majority of people. This approach to improving HIV treatment successfully reduced the time from diagnosis to ART initiation from 264 days in 2015 to 23 days in 2019 [11], which contributed to viral suppression rates (viral load <200 copies/mL) among those diagnosed reaching nearly 90% by mid-2019. GECHO also included intensive contact tracing to identify new cases.

In this study, we use mathematical modeling to evaluate the impact of the systematic expansion of HIV testing for PWID, and the GECHO treatment and contact tracing interventions. We refer to these interventions collectively as GECHO+, meaning GECHO plus enhanced testing.

## METHODS

### The Model

**Figure 1.**
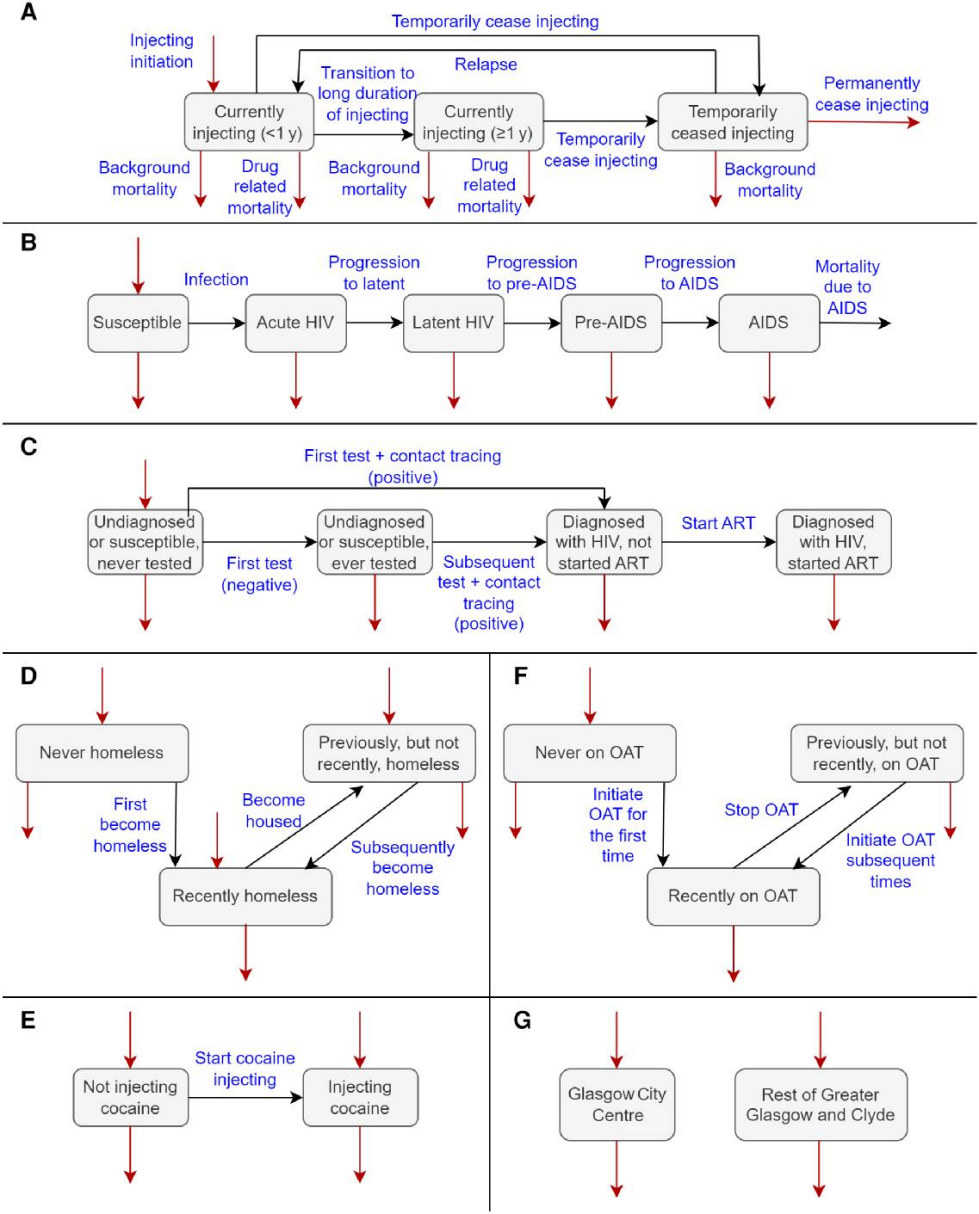
Model schematics: injecting status (*A*), human immunodeficiency virus (HIV) progression (*B*), testing and treatment (*C*), homelessness (*D*), cocaine injecting (*E*), opioid agonist therapy (*F*), and geography (*G*). Red arrows illustrate how people who inject drugs (PWID) are recruited into and leave the model. Note that non-HIV mortality and permanent cessation of injecting have been explicitly labeled in (*A*), whereas red outflow arrows in (*B–G*) refer to combined background mortality, drug-related mortality, and permanent cessation of injecting. The total number of PWID diagnosed with HIV who have not yet been asked about contacts is also tracked to inform the contact tracing rate. Abbreviations: ART, antiretroviral therapy; HIV, human immunodeficiency virus; OAT, opioid agonist therapy.

We constructed a dynamic, deterministic, compartmental model of HIV transmission among PWID. The population is stratified by injecting status and duration, HIV progression, diagnosis and treatment status, homelessness, cocaine injecting, OAT status, and geographical region; see model schematics illustrated in Figure 1.

PWID enter the model when they initiate injecting drug use. They enter as susceptible to HIV acquisition, never tested for HIV, and not accessing OAT. They may enter in any homelessness or cocaine injecting state and into either GCC or RoGGC. PWID leave the model through background mortality, drug-related mortality (which has substantially increased in Scotland over the period of the outbreak [12]), AIDS-related mortality, or permanent cessation of injecting.

PWID can transition between currently injecting and temporarily ceased injecting. Currently injecting PWID are defined to be those who have injected in the last 6 months and are categorized by the duration of their current injecting period (<1 year/≥1 year) due to the association between duration of injecting period and temporary cessation [13]. When temporarily ceased, PWID may relapse, or leave the model by permanently ceasing injecting. We assume PWID on OAT are more likely to temporarily cease injecting [13], unless they inject cocaine.

In the model, once HIV is acquired, PWID progress through the HIV infection stages (acute/latent/pre-AIDS/AIDS). PWID on ART experience slower HIV progression and reduced AIDS mortality. PWID acquire HIV at a rate dependent on the number of PWID in each infection stage, the proportion of PWID living with HIV accessing ART, the proportions of PWID with risk factors for HIV acquisition and transmission (homelessness/cocaine injecting), and the proportion accessing OAT. HIV transmission varies by infection stage [14], and is higher for those who are homeless and/or injecting cocaine but reduced if on OAT. The effectiveness of ART at preventing transmission improves throughout the outbreak as a greater proportion of PWID become virally suppressed. We assume partial assortative population mixing based on homelessness, cocaine injecting, and geography.

PWID can be tested for HIV either through systematic testing, with different rates depending on first/subsequent test, or contact tracing. Systematic testing rates increase over time and are dependent on homelessness and OAT, to account for the targeted testing interventions in homeless services and pharmacies respectively, and geographical region. Contact tracing moves PWID with undiagnosed HIV into the “diagnosed” category. We assume that contact tracing does not necessarily lead to engagement with systematic testing services, and so susceptible PWID tested through contact tracing are not moved from "never" to "ever" tested. Further details about the implementation of contact tracing in the model are described in the Supplementary Material.

PWID diagnosed with HIV initiate ART at a time-dependent rate to reflect the improvements made to the time from HIV diagnosis to treatment initiation throughout the outbreak. Attrition from ART is not included in the model due to the high levels of viral suppression among PWID diagnosed as part of the outbreak [11, 15] and the short time scale being modeled.

PWID can move from being “never” homeless to “recently homeless,” defined as those who have been homeless in the last 6 months. Those who have been “recently homeless” can move between the “recently homeless” and “previously but not recently homeless” compartments. Different rates are used for becoming homeless first and subsequent times to capture patterns in the data relating to the proportions of PWID who have ever been homeless or been homeless in the last 6 months [16]. A similar structure is used for the OAT component, as shown in Figure 1, with “recently on OAT” defined as being on OAT in the last 6 months. The rates for becoming homeless depend on geography.

In the model, PWID can start injecting cocaine, but cannot leave the “injecting cocaine” compartment, unless they stop injecting entirely. They may temporarily cease, but those who relapse will continue in the “injecting cocaine” compartment. This is because the time frame where cocaine injecting started increasing in Glasgow (2015–2020) [16] is relatively short. The rate for initiating cocaine injecting varies over time and by geographical region to account for the increase in cocaine injecting over the course of the outbreak, which was particularly marked in GCC.

The model does not account for PWID movement between GCC and RoGGC due to the short time scale being modeled.

Submodels were used to calibrate injecting cessation and relapse rates; further information can be found in the Supplementary Material.

### Parameterization and Calibration

Most of the data used to parameterize and calibrate the model came from the biennial cross-sectional Needle Exchange Surveillance Initiative (NESI) surveys [16] undertaken among PWID in contact with services. Data collected relate to injecting behavior, use of harm reduction services, BBVs, and other drug-related health harms. The participants complete a questionnaire administered by an interviewer and are asked to provide a voluntary dried blood spot sample, which is used to test for the presence of BBV markers, including HIV. The modeled population reflects the population of PWID participating in this survey, who are likely at higher risk of BBV transmission than the wider group of people who have ever injected drugs. Public Health Scotland and National Health Service Greater Glasgow and Clyde provided data on annual numbers of HIV diagnoses (from the data sources used to produce the “HIV in Scotland” update [17]), annual numbers of HIV tests in drug services [18], and data related to viral suppression and ART [11, 15] within GGC. Literature sources were used for the remaining parameters.

There is inconsistency in the data regarding estimated PWID population sizes in GCC [19], the HIV prevalence from NESI [16], and the reported number of diagnoses [17], with the population size estimates and HIV prevalence suggesting there should have been more diagnoses. As the population participating in NESI is likely to be at higher risk of BBV transmission, and this is the population we wish to model, we have allowed a wide prior distribution for the population size estimate so it calibrates to fit the HIV prevalence and diagnosis data. Further details can be found in the Discussion and in the Supplementary Material.

We implemented an approximate Bayesian computation sequential Monte Carlo algorithm [20] to calibrate the model parameters to data relating to HIV prevalence, HIV diagnoses, history of HIV testing, prevalence of homelessness and cocaine injecting, and coverage of OAT. The algorithm was run with 7500 parameter sets to give 7500 model fits, which were used to give the median and 95% credible interval (CrI; 2.5th to 97.5th percentile range) for all model projections. Further details, including specifics about the model fit, are provided in the Supplementary Material.

Table 1 provides information on the data used to parameterize and calibrate the aspects of the model relating to the interventions. Full details about the model parameters and calibration data are provided in Supplementary Tables 1 and 2.

**Table 1.** Data Used to Inform How the Interventions Are Modeled

Description	Value	Source	Notes	Posteriors: Median (95% CrI)
Time from HIV diagnosis to ART initiation (days) by year	2015: 264 (Q1–Q3: 94–556) 2016: 139 (Q1–Q3: 49–280) 2017: 73 (Q1–Q3: 25–156) 2018: 28 (Q1–Q3: 18–51) 2019: 23 (Q1–Q3: 12–38)	[11, 15]	Included in model as parameter	2015: 298 (198–406) 2016: 143 (93.9–193) 2017: 75.2 (45.0–111) 2018: 28.0 (22.3–34.8) 2019: 23.3 (17.0–30.8)
Proportion of HIV-positive PWID on ART who are virally suppressed	Pre-mid-2016: Range, 0.61–0.68 Post-mid-2018: Range, 0.84–0.89	[11, 15]	Included in model as parameter	Pre: 0.65 (.62–.67) Post: 0.87 (.85–.89)
No. of HIV tests carried out in drug services in GGC by year	2013: 746 2014: 912 2015: 1673 2016: 2616 2017: 2308 2018: 3610 2019: 4939	West of Scotland Specialist Virology Centre [18]	Calibrating model parameters to between 50% and 100% of these data values (see Discussion for more details)	2013: 648 (422–905) 2014: 648 (422–907) 2015: 981 (740–1310) 2016: 1640 (1240–2250) 2017: 2070 (1550–2810) 2018: 2400 (1760–3270) 2019: 2740 (1960–3780)
Proportion who have ever had an HIV test among PWID who have never been homeless	Pre: 0.69 (95% CI, .62–.76) Early: 0.81 (95% CI, .76–.87) Mid: 0.78 (95% CI, .71–.85) Late: 0.84 (95% CI, .79–.89)	NESI [16]	Calibrating model parameters to these data	Pre: 0.74 (.70–.79) Early: 0.75 (.69–.80) Mid: 0.80 (.76–.84) Late: 0.84 (.79–.88)
Proportion who have ever had an HIV test among PWID who have previously been homeless but are not recently homeless	Pre: 0.83 (95% CI, .79–.87) Early: 0.89 (95% CI, .86–.93) Mid: 0.93 (95% CI, .90–.96) Late: 0.95 (95% CI, .92–.97)	NESI [16]	Calibrating model parameters to these data	Pre: 0.84 (.81–.87) Early: 0.87 (.84–.90) Mid: 0.92 (.90–.94) Late: 0.95 (.93–.96)
Proportion who have ever had an HIV test among PWID who are recently homeless	Pre: 0.81 (95% CI, .76–.87) Early: 0.86 (95% CI, .80–.91) Mid: 0.91 (95% CI, .87–.96) Late: 0.92 (95% CI, .89–.96)	NESI [16]	Calibrating model parameters to these data	Pre: 0.80 (.76–.84) Early: 0.84 (.80–.87) Mid: 0.90 (.87–.91) Late: 0.92 (.90–.94)
Proportion who have ever had an HIV test among PWID who have never been on OAT	Pre: 0.36 (95% CI, .16–.56) Early: 0.38 (95% CI, .21–.55) Mid: 0.41 (95% CI, .24–.59) Late: 0.51 (95% CI, .38–.64)	NESI [16]	Calibrating model parameters to these data	Pre: 0.31 (.25–.37) Early: 0.33 (.27–.39) Mid: 0.42 (.37–.48) Late: 0.48 (.42–.54)
Proportion who have ever had an HIV test among PWID who have previously been on OAT but are not recently on OAT	Pre: 0.77 (95% CI, .62–.93) Early: 0.83 (95% CI, .64–1.00) Mid: 0.93 (95% CI, .84–1.00) Late: 0.88 (95% CI, .76–1.00)	NESI [16]	Calibrating model parameters to these data	Pre: 0.87 (.84–.89) Early: 0.89 (.86–.91) Mid: 0.94 (.92–.95) Late: 0.96 (.95–.97)
Proportion who have ever had an HIV test among PWID who are recently on OAT	Pre: 0.81 (95% CI, .78–.84) Early: 0.89 (95% CI, .86–.91) Mid: 0.92 (95% CI, .90–.95) Late: 0.95 (95% CI, .93–.97)	NESI [16]	Calibrating model parameters to these data	Pre: 0.85 (.82–.88) Early: 0.88 (.85–.90) Mid: 0.93 (.91–.94) Late: 0.95 (.94–.96)
Proportion of HIV diagnoses made in drugs services	2014: 0.22 (95% CI, .03–.54) 2015: 0.39 (95% CI, .28–.48) 2016: 0.39 (95% CI, .26–.52) 2017: 0.31 (95% CI, .20–.42) 2018: 0.41 (95% CI, .23–.58) 2019: 0.32 (95% CI, .17–.49) 2020: 0.53 (95% CI, .33–.67)^a^	Estimate derived from proportion of HIV diagnoses in drugs services and prisons [17], and proportion of HIV tests carried out in drugs services vs drugs services and prisons [18]	Calibrating model parameters to these data	2014: 0.34 (.21–.47) 2015: 0.40 (.29–.48) 2016: 0.47 (.39–.52) 2017: 0.50 (.45–.55) 2018: 0.52 (.47–.56) 2019: 0.53 (.49–.57)
In response to the outbreak, people diagnosed with HIV asked about contacts within approximately 1 mo of diagnosis	398 unique contacts reported from 184 HIV cases, of whom 150/398 (38%) had HIV (includes 2020 data)	Personal correspondence relating to [11]	NA	NA

Outbreak eras are defined as follows: Pre, 2013–2014; Early, 2015–2016; Mid, 2017–2018; Late, 2019–2020.

Abbreviations: ART, antiretroviral therapy; CI, confidence interval; CrI, credible interval; GGC, Greater Glasgow and Clyde; HIV, human immunodeficiency virus; NA, not applicable; NESI, Needle Exchange Surveillance Initiative; OAT, opioid agonist therapy; PWID, people who inject drugs; Q1, quartile 1; Q3, quartile 3.

^a^Included to show general trend over time.

### Estimating the Impact of the Interventions

All calibrated parameter sets are used to investigate the impact of the interventions on the outbreak. The time horizon for the analysis spans from the beginning of 2015 to the beginning of 2020. We compare the GECHO+ scenario, which was calibrated to data on the outbreak, with multiple counterfactual scenarios where improvements in testing/treatment interventions are absent (scenarios R1, R2, R3) or implemented earlier (scenarios I1, I2, I3, P1). The scenarios and their definitions are listed in Table 2. For each of these scenarios, we calculate the prevalence and incidence at the beginning of 2015 and 2020. We also calculate the relative increase in prevalence and incidence for each scenario at the beginning of 2020 compared to GECHO+, the annual number of new HIV cases and the total number of excess cases for each scenario.

**Table 2.** Definitions for Each Scenario Investigated

Scenario	Details
S0: GECHO+	Testing and treatment interventions are modeled as they actually happened.
R1: Removing HIV testing and treatment improvements	Combination of R2 and R3 described below.
R2: Removing improvements in HIV treatment	The time from diagnosis to treatment initiation is set to the 2015 value for all years. The proportion of PWID diagnosed with HIV achieving viral suppression is fixed to the pre/early outbreak value.
R3: Removing improvements in HIV testing	The increase in testing rates during the outbreak is set to 0, so that testing rates remain at pre-outbreak levels. The homeless and OAT effects on testing are set to their pre-outbreak values. The contact tracing rate is set to zero.
I1: Immediate improvements in HIV testing and treatment interventions	Combination of I2 and I3 below.
I2: Immediate improvement in HIV treatment	From the beginning of 2015, the time from diagnosis to treatment initiation is increased from the 2015 value to the 2019 value over the course of 1 y.
I3: Immediate improvement in HIV testing	The full increase in testing rates during the outbreak is set to occur immediately. From the beginning of 2015, the testing rate increases from the pre-outbreak value to the late-outbreak value over the course of 1 y. Targeted testing toward those who are homeless and/or on OAT is adjusted to start at the beginning of 2015.
P1: Preemptive improvements in HIV testing and treatment interventions	The time from diagnosis to treatment initiation is set to the 2019 value for all time. Testing rates are set to their highest value for all time. Targeted testing interventions for those who are homeless and/or on OAT are set to start before the modeled time period. The contact tracing rate is set to zero because this intervention is typically implemented in response to an outbreak instead of being in place permanently.

Outbreak eras are defined as follows: Pre, 2013–2014; Early, 2015–2016; Mid, 2017–2018; Late, 2019–2020.

Abbreviations: GECHO+, Glasgow Enhanced Care HIV Outreach plus enhanced testing; HIV, human immunodeficiency virus; OAT, opioid agonist therapy; PWID, people who inject drugs.

Sensitivity analysis methods and results are provided in the Supplementary Material.

All coding and analysis was performed in MATLAB.

## RESULTS

**Figure 2.**
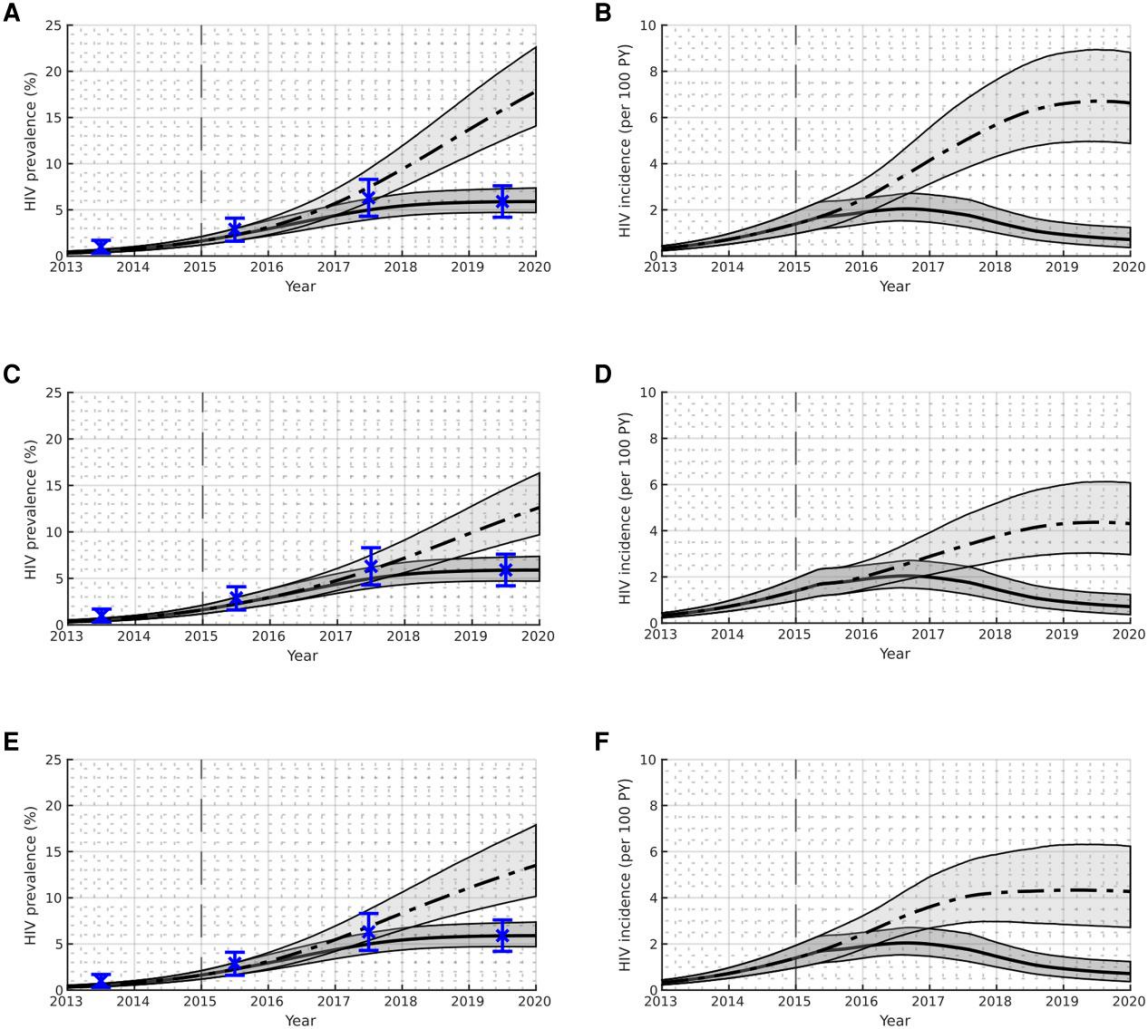
Human immunodeficiency virus prevalence and incidence among current people who inject drugs for counterfactual scenarios where interventions have been removed (R1, R2, R3); see Table 2 for scenario definitions. The Glasgow Enhanced Care HIV Outreach plus enhanced testing scenario (S0) is illustrated using dark gray shading and a solid black line to indicate the median; the counterfactual scenarios are illustrated using light gray shading and a dashed black line to indicate the median. The boundaries of the shading indicate the 95% credible intervals. Validation data are illustrated in blue. Abbreviations: HIV, human immunodeficiency virus; PY, person-years.

**Figure 3.**
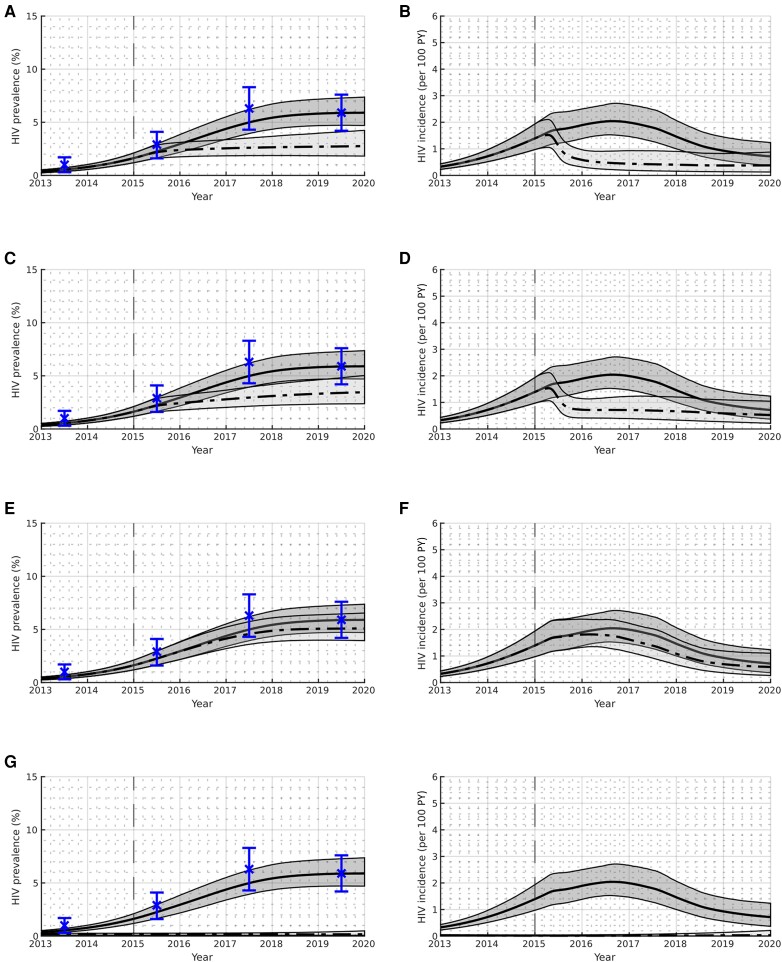
Human immunodeficiency virus prevalence and incidence among current people who inject drugs for counterfactual scenarios where interventions are implemented immediately on detection of the outbreak (I1, I2, I3) or prior to the start of the outbreak (P1); see Table 2 for scenario definitions. The Glasgow Enhanced Care HIV Outreach plus enhanced testing scenario (S0) is illustrated using dark gray shading and a solid black line to indicate the median; the counterfactual scenarios are illustrated using light gray shading and a dashed black line to indicate the median. The boundaries of the shading indicate the 95% credible intervals. Validation data are illustrated in blue. Abbreviations: HIV, human immunodeficiency virus; PY, person-years.

Results for each of the modeled scenarios are presented in Tables 3–5 and illustrated in Figures 2 and 3.

**Table 3.** Prevalence Results

Scenario	Prevalence in 2015, %	Prevalence in 2020, %	Relative Increase in Prevalence From S0 in 2020 (%)
S0: GECHO+	1.6 (1.2–2.1)	5.9 (4.7–7.4)	NA
R1: Removing HIV testing and treatment improvements	1.6 (1.2–2.1)	17.8 (14.1–22.6)	201.7 (147.6–279.6)
R2: Removing improvements in HIV treatment	1.6 (1.2–2.1)	12.6 (9.7–16.3)	113.1 (76.3–164.9)
R3: Removing improvements in HIV testing	1.6 (1.2–2.1)	13.5 (10.2–17.9)	128.1 (82.3–195.0)
I1: Immediate improvements in HIV testing and treatment interventions	1.6 (1.2–2.1)	2.8 (1.8–4.2)	−53.0 (−66.4 to −36.4)
I2: Immediate improvement in HIV treatment	1.6 (1.2–2.1)	3.5 (2.4–5.0)	−41.0 (−55.3 to −25.8)
I3: Immediate improvement in HIV testing	1.6 (1.2–2.1)	5.1 (3.9–6.5)	−13.5 (−22.5 to −7.3)
P1: Preemptive improvements in HIV testing and treatment interventions	0.2 (.1–.3)	0.2 (.1–.5)	−97.0 (−98.8 to −92.3)

Values given are the median (95% credible interval).

Abbreviations: GECHO+, Glasgow Enhanced Care HIV Outreach plus enhanced testing; HIV, human immunodeficiency virus; NA, not applicable.

**Table 4.** Incidence Results

Scenario	Incidence in 2015 per 100 PY	Incidence in 2020 per 100 PY	Relative Increase in Incidence From S0 in 2020, %	Peak Incidence per 100 PY	Relative Increase in Peak Incidence From S0, %
S0: GECHO+	1.4 (1.0–1.9)	0.7 (.4–1.2)	NA	2.1 (1.6–2.8)	NA
R1: Removing HIV testing and treatment improvements	1.4 (1.0–1.9)	6.6 (4.9–8.8)	832.0 (469.7–1571.2)	6.8 (5.0–9.0)	222.1 (131.5–346.0)
R2: Removing improvements in HIV treatment	1.4 (1.0–1.9)	4.3 (3.0–6.1)	500.9 (294.7–936.7)	4.4 (3.1–6.2)	109.7 (47.8–197.0)
R3: Removing improvements in HIV testing	1.4 (1.0–1.9)	4.3 (2.7–6.2)	496.0 (260.1–972.3)	4.5 (3.1–6.4)	111.1 (50.2–213.2)
I1: Immediate improvements in HIV testing and treatment interventions	1.4 (1.0–1.9)	0.4 (.1–.9)	−46.9 (−69.5 to −22.9)	1.5 (1.1–2.1)	−26.2 (−49.3 to −8.8)
I2: Immediate improvement in HIV treatment	1.4 (1.0–1.9)	0.5 (.2–1.0)	−26.5 (−46.9 to −8.5)	1.5 (1.1–2.1)	−25.7 (−48.6 to −8.2)
I3: Immediate improvement in HIV testing	1.4 (1.0–1.9)	0.6 (.3–1.1)	−17.4 (−33.4 to −7.4)	1.9 (1.4–2.5)	−10.0 (−22.8 to −.6)
P1: Preemptive improvements in HIV testing and treatment interventions	0.0 (.0–.0)	0.0 (.0–.2)	−95.3 (−98.7 to −79.6)	0.1 (.0–.2)	−97.5 (−98.9 to −89.8)

Values given are the median (95% credible interval).

Abbreviations: GECHO+, Glasgow Enhanced Care HIV Outreach plus enhanced testing; HIV, human immunodeficiency virus; NA, not applicable; PY, person-years.

**Table 5.** New Cases Results

Scenario	New Cases in 2015	New Cases in 2016	New Cases in 2017	New Cases in 2018	New Cases in 2019	Total New Cases	Total Excess Cases
S0: GECHO+	32 (22–45)	38 (28–50)	34 (23–47)	22 (14–33)	16 (8–26)	142 (105–190)	NA
R1: Removing HIV testing and treatment improvements	35 (25–50)	62 (45–87)	94 (68–136)	119 (86–173)	127 (91–184)	437 (327–612)	294 (213–441)
R2: Removing improvements in HIV treatment	32 (23–45)	46 (33–64)	63 (44–93)	78 (53–116)	83 (56–124)	303 (220–429)	160 (104–255)
R3: Removing improvements in HIV testing	35 (24–50)	58 (42–80)	75 (53–111)	81 (54–127)	82 (52–131)	332 (237–481)	189 (118–311)
I1: Immediate improvements in HIV testing and treatment interventions	20 (14–29)	9 (5–17)	8 (3–17)	7 (3–17)	7 (2–17)	53 (30–92)	−87 (−125 to −58)
I2: Immediate improvement in HIV treatment	21 (15–30)	13 (8–22)	13 (7–23)	12 (6–22)	11 (5–21)	71 (43–112)	−69 (−102 to −44)
I3: Immediate improvement in HIV testing	31 (22–44)	33 (24–45)	26 (17–38)	16 (9–27)	12 (6–22)	120 (86–164)	−21 (−37 to −11)
P1: Preemptive improvements in HIV testing and treatment interventions	0 (0–1)	0 (0–1)	0 (0–1)	0 (0–2)	1 (0–4)	2 (0–9)	−139 (−185 to −103)

Values given are the median (95% credible interval). A negative value for excess cases indicates that cases were averted rather than in excess.

Abbreviations: GECHO+, Glasgow Enhanced Care HIV Outreach plus enhanced testing; HIV, human immunodeficiency virus; NA, not applicable.

### GECHO+

In line with the observed public health surveillance data, Figures 2 and 3 show that under the GECHO+ scenario (S0), the HIV prevalence increased from 1.6% (95% CrI, 1.2%–2.1%) at the beginning of 2015 to 5.9% (95% CrI, 4.7%–7.4%) by the beginning of 2020. The modeling suggests that incidence peaked around 2016 at a value of 2.1/100 person-years (PY) (95% CrI, 1.6–2.8) before declining to 0.7/100 PY (95% CrI, .4–1.2) by the beginning of 2020, with 142 (95% CrI, 105–190) cases in the years 2015–2019 inclusive.

### Impact of the Outbreak Response

If the GECHO+ improvements in testing and treatment had not been implemented (R1), the modeling suggests that HIV prevalence would be 201.7% (95% CrI, 147.6%–279.6%) greater by 2020 compared to the GECHO+ scenario, likely exceeding 14.1%, and still increasing. Incidence would be 832.0% (95% CrI, 469.7%–1571.2%) greater by 2020 and is unlikely to have peaked by this point, reaching a value of 6.6/100 PY (95% CrI, 4.9–8.8), as illustrated in Figure 2*B*. Table 5 suggests there would have been 294 (95% CrI, 213–441) additional infections between 2015 and 2020 if GECHO+ had not been implemented, an increase of 210.1% (95% CrI, 154.4%–292.2%).

Breaking this down, we can see that without the improvements in HIV treatment (R2), HIV prevalence would be 113.1% (95% CrI, 76.3%–164.9%) greater by 2020 and incidence would be 500.9% (95% CrI, 294.7%–936.7%) greater. Over the study period, there would have been 160 (95% CrI, 104–255) additional infections compared to the GECHO+ scenario, an increase of 113.9% (95% CrI, 76.4%–167.4%). The outbreak would have had a similar profile to the scenario where both testing and treatment improvements are removed (R1), with HIV prevalence continuing to increase throughout the study period, and incidence unlikely to peak before the end of 2019, as shown in Figure 2*D*.

Without the improvements in HIV testing (R3), HIV prevalence would be 128.1% (95% CrI, 82.3%–195.0%) greater and incidence would be 496.0% (95% CrI, 260.1%–972.3%) greater by 2020. The modeling suggests there would have been 189 (95% CrI, 118–311) more infections without this intervention, an increase of 134.4% (95% CrI, 87.0%–205.5%). Again, we see a similar outbreak profile to the case where neither testing nor treatment improvements are accounted for.

### Impact of Intervening Earlier

If the GECHO+ improvements in testing and treatment had been implemented to their peak capacity immediately in 2015 (I1), when the outbreak was first detected, then Figure 3*A* and 3*B* suggests the outbreak could have been immediately reduced and controlled. The prevalence would have plateaued, reaching a value of 2.8% (95% CrI, 1.8%–4.2%) by the beginning of 2020, which is 53.0% (95% CrI, 36.4%–66.4%) lower than the GECHO+ scenario. The incidence would have peaked in 2015, at a value 26.2% (95% CrI, 8.8%–49.3%) lower compared to the GECHO+ scenario, before rapidly returning to pre-outbreak levels. The modeling suggests there would have been 87 (95% CrI, 58–125) fewer infections, a 62.7% (95% CrI, 43.6%–76.6%) reduction.

A similar situation is shown in Figures 2*C* and 3*D* when we consider what would have happened if only the treatment interventions had been scaled up immediately (I2), with prevalence reaching 3.5% (95% CrI, 2.4%–5.0%) by the beginning of 2020, which is 41.0% (95% CrI, 25.8%–55.3%) lower than the GECHO+ scenario. The decline in incidence also follows a similar pattern, with 69 (95% CrI, 44–102) fewer infections over the modeled time period, which is a decrease of 49.5% (95% CrI, 31.9%–64.8%).

However, when we consider the situation when only the improvements in testing are implemented immediately (I3), and the improvements in treatment follow their historical course, the outbreak shows a similar profile to the GECHO+ scenario. The prevalence by the beginning of 2020 is only 13.5% (95% CrI, 7.3%–22.5%) lower, and the incidence is 17.4% (95% CrI, 7.4%–33.4%) lower, with 21 (95% CrI, 11–37) fewer infections corresponding to a 15.0% (95% CrI, 8.2%–24.8%) decrease.

Figure 3 *E* and 3*F* shows that if the testing and treatment interventions, excluding contact tracing, had already been in place prior to the outbreak beginning (P1), then the outbreak could have been avoided.

## DISCUSSION

We use a mathematical modeling approach to evaluate the impact of the GECHO+ intervention in response to an HIV outbreak among PWID, which consisted of a novel HIV care outreach approach to treating PWID living with HIV and the scale-up of HIV testing among PWID. Our results show that GECHO+ was effective at bringing the outbreak under control by the beginning of 2020, likely preventing hundreds of infections. If the improvements to the interventions had been implemented to their peak capacity upon detection of the outbreak in 2015, the outbreak would have been immediately controlled. Furthermore, the outbreak could have been avoided if the interventions had been at peak capacity in the years before 2015.

A key strength of this model is that it was fitted to rich epidemiological data using Bayesian methods that account for uncertainty in model parameters.

However, there are limitations to consider. First, we use a deterministic, compartmental model because these models are flexible, allowing us to use a variety of data types to account for multiple features of the outbreak and population, and are computationally feasible even for large model complexity. However, these models do not account for network structure in the population, which may impact the epidemic profile and estimated impact of interventions [21, 22]. Therefore, it may be appropriate for further modeling of this outbreak to utilize phylogenetic analysis of the outbreak [6], particularly for assessing network-based interventions such as contact tracing.

Second, the majority of the outbreak-specific data used to parametrize and calibrate the model come from the NESI surveys. These surveys may have a greater response rate in GCC compared to RoGGC. Furthermore, by recruiting from sites supplying injecting equipment, the surveys target individuals who are actively injecting and represent PWID with higher HIV risk compared to those in recovery or on long-term OAT. This may contribute to the observed inconsistency between the data regarding HIV prevalence [16], HIV diagnoses [17], and historical estimates for the PWID population size in Glasgow [19]. As we are modeling a higher-risk population than is reflected in the PWID population size estimates, we calibrated the population size using a wide uninformed prior, which results in a smaller population size compared to the data estimates. Assuming a smaller population size would impact the ratio of HIV tests per person. To account for this, we adjusted the metric used to compare the annual number of HIV tests to data in the calibration process; model predictions within 50%–100% of the data value are considered an equally good fit. This leads to wider posterior distributions for the testing rates. This uncertainty propagates to the model results and is reflected in the reported credible intervals.

Third, certain features of the outbreak were necessarily excluded to minimize model complexity. For example, sexual transmission was not explicitly modeled because high prevalence of hepatitis C virus among the outbreak cohort suggests transmission occurred mainly via sharing of injecting equipment [6]. Incarceration dynamics were not included because the data from NESI suggest that cocaine and homelessness are more important risk factors to include [5, 16]. The impact of NSPs was not explicitly included in the model because provision remained relatively stable over the course of the outbreak [5, 16].

Fourth, though we have detailed data for the number of HIV tests carried out in drugs services, there were diagnoses among PWID in the outbreak cohort made in other locations, where fewer data are available specifically relating to PWID. We were able to use the 2019–2020 NESI survey [16] to estimate the proportion of HIV tests carried out in other locations at 1 time point, but relied on approximate estimates for the proportion of HIV diagnoses made in drugs services to estimate how this changed over time. Further details are provided in the Supplementary Material.

Finally, the modeling does not extend past the beginning of 2020; therefore, we cannot evaluate the longer-term impact of the interventions on the outbreak. A key reason for this is that that the coronavirus disease 2019 (COVID-19) pandemic reached the United Kingdom in 2020, which disrupted interventions and data collection [23]. Further data are required to understand the impact of COVID-19 on the HIV outbreak.

Previous modeling work also demonstrates the benefits of testing and treatment interventions in combination. Gonsalves et al [24] modeled the impact of testing, treatment, and harm reduction interventions on an HIV outbreak that began in Scott County, United States, in 2014. Some modeled scenarios suggest that earlier intervention could have had a substantial impact on the outbreak, preventing it from happening. The modeling by Flountzi et al [25] of the 2012–2013 HIV outbreak in Athens, Greece, demonstrated the beneficial impact of a combined harm reduction and targeted testing/care outreach intervention. Cepeda et al [26] found a beneficial impact of combined harm reduction and treatment interventions when HIV is increasing among PWID.

## CONCLUSIONS

The HIV outbreak in Glasgow occurred under the backdrop of moderate to high levels of OAT and NSP coverage, alongside an increase in cocaine injecting and initially suboptimal HIV testing and treatment interventions.

The GECHO clinical outreach approach to improving HIV treatment in response to the outbreak managed to achieve viral suppression in nearly 90% of PWID diagnosed with HIV by mid-2019, a large improvement compared to 2015 where no more than 40% were virally suppressed. In combination with increased HIV testing (GECHO+), the modeling suggests these interventions brought the outbreak under control before 2020. However, our results highlight the importance of a combined approach; improvements in either testing or treatment alone would not have controlled the outbreak.

HIV testing and treatment interventions should be maintained even when HIV apparently is endemically low, as had been the case prior to the outbreak in GGC. Given the financial pressures facing the healthcare system, analysis of the cost-effectiveness of GECHO+ should be carried out as soon as possible to understand the health economic benefit of a rapid clinical intervention at the outset.

## Supplementary Data

Supplementary materials are available at *The Journal of Infectious Diseases* online (http://jid.oxfordjournals.org/). Supplementary materials consist of data provided by the author that are published to benefit the reader. The posted materials are not copyedited. The contents of all supplementary data are the sole responsibility of the authors. Questions or messages regarding errors should be addressed to the author.

## Supplementary Material

jiae206_Supplementary_Data

## Notes

***Author contributions.*** L. I. A., H. F., P. V., M. H., and S. J. H. conceived the study. L. I. A., H. F., J. S., and P. V. contributed to model design. L. I. A., supervised by H. F., J. S., P. V., M. H., and S. J. H., performed all the modeling and undertook all formal analysis. S. J. H., A. M., K. M. A. T., R. M., and S. E. P. provided the survey and other epidemiological data to parameterize the model. Analysis of the survey and other epidemiological data that informed the model was performed by L. I. A., K. M. A. T., and R. M. All authors interpreted the findings. L. I. A. wrote the first and subsequent drafts of the manuscript and all remaining authors contributed to critical review of the manuscript.

***Acknowledgments.*** We acknowledge the contributions of Julie Craik and Daniel Carter, who provided data for model parameterization and facilitated interpretation of the findings.

***Data sharing.*** Supplementary Material relating to this study are available online, which include the data used to parameterize and calibrate the model. Aggregate-level survey data are available at https://publichealthscotland.scot/publications/needle-exchange-surveillance-initiative-nesi/needle-exchange-surveillance-initiative-nesi/. Additional patient-level data can be sourced through consultation with NHS Greater Glasgow and Clyde. Model code will be made available immediately following publication. The code will be shared with researchers who provide a methodologically sound proposal approved by L. I. A. and P. V. Proposals should be directed to lara.gordon.2019@bristol.ac.uk and peter.vickerman@bristol.ac.uk; requesters will need to sign a data access agreement.

***Financial support*.** This work was supported in part by grant MR/N0137941/1 for the GW4 BIOMED Medical Research Council (MRC) doctoral training partnership, awarded to the Universities of Bath, Bristol, Cardiff, and Exeter from the MRC/UK Research and Innovation. This study was also supported in part by Public Health Scotland and the National Institute for Health and Care Research (NIHR). NIHR funding was through the Programme Grants for Applied Research (Evaluating the Population Impact of Hepatitis C Direct Acting Antiviral Treatment as Prevention for People Who Inject Drugs (EPIToPe) grant number RP-PG-0616–20 008) and NIHR Health Protection Research Unit (HPRU) in Behavioural Science and Evaluation (NIHR200877). The intervention evaluated and associated administrative data used were funded by NHS Greater Glasgow and Clyde. The Needle Exchange Surveillance Initiative was funded by Public Health Scotland and NIHR EPIToPe. H. F., P. V., and M. H. acknowledge support from the NIHR HPRU in Evaluation of Interventions and Behavioural Science. J. S. and P. V. acknowledge funding from the Wellcome Trust (WT 226619/Z/22/Z). This work was carried out using the computational facilities of the Advanced Computing Research Centre, University of Bristol (http://www.bristol.ac.uk/acrc/). For the purpose of open access, the authors have applied a Creative Commons Attribution (CC BY) license to any Author Accepted Manuscript version arising.
