# Supplementary material for: Testing and Treatment Interventions in Community Settings Key to Controlling a Recent Human Immunodeficiency Virus Outbreak Among People Who Inject Drugs in Glasgow: A Modeling Study
Source: J Infect Dis. 2024 May 22;230(1):e48–59. doi: 10.1093/infdis/jiae206 (PMC11272080; doi:10.1093/infdis/jiae206)
Supplement: jiae206_Supplementary_Data [file jiae206_supplementary_data.pdf]

Supplementary material for 'Testing and Treatment Interventions in Community Settings Key to Controlling a Recent Human Immunodeficiency Virus Outbreak Among People Who Inject Drugs in Glasgow: A Modeling Study'

Authors: Lara I Allen*, Hannah Fraser, Jack Stone, Andrew McAuley, Kirsten MA Trayner, Rebecca Metcalfe, S Erica Peters, Sharon J Hutchinson**, Peter Vickerman**, Matthew Hickman**

* corresponding author ** joint senior authors

Contents

1 Model description **2**
1.1 Main model . . . 2
1.2 Submodels . . . 3

2 Model equations **4**
2.1 Preliminaries . . . 4
2.2 Leaving the model . . . 5
2.3 Recruitment . . . 5
2.4 Injecting status . . . 6
2.5 Infection stage/transmission . . . 6
2.6 Testing and treatment for HIV . . . 10
2.7 Homelessness . . . 15
2.8 Cocaine injecting . . . 15
2.9 OAT . . . 16
2.10 Geography . . . 17
2.11 Submodel equations . . . 17
2.12 Additional model details . . . 18

3 Parameterisation and calibration **20**
3.1 The calibration algorithm . . . 20
3.2 Further calibration details . . . 20
3.3 Parameter and calibration data tables . . . 22

4 Model fit **34**

5 Sensitivity Analysis **37**
5.1 Methods . . . 37
5.2 Findings . . . 37
5.3 Discussion . . . 37

References **46**

1 Model description

Before providing full model details, including equations, we will first outline the key features of the model.

1.1 Main model

We constructed a dynamic, deterministic, compartmental model of HIV transmission among people who inject drugs (PWID). The population is stratified by injecting status (currently injecting (< 1 yr)/currently injecting (≥ 1 yr)/temporarily ceased injecting), HIV progression (susceptible/infected (acute)/infected (latent)/infected (pre-AIDS)/infected (AIDS)), diagnosis and treatment status (undiagnosed or susceptible and never tested/undiagnosed or susceptible and ever tested/diagnosed and not started antiretrovial treatment (ART)/diagnosed and started ART), homelessness (never/previously/recently), cocaine injecting (never/ever), opioid agonist therapy (OAT) status (never/previously/recently) and geographical region (accessing services in Glasgow City Centre (GCC)/Rest of Greater Glasgow and Clyde (RoGGC)). Schematics are illustrated in the main text. PWID enter the model as currently injecting, susceptible to HIV, never tested for HIV and not accessing OAT. They may enter in any homelessness or cocaine injecting state and into either GCC or RoGGC. PWID leave the model through background mortality, drug related mortality (which has substantially increased in Scotland over the period of the outbreak [16]), AIDS related mortality or permanent cessation of injecting.

PWID can transition between currently injecting and temporarily ceased injecting. Currently injecting PWID are defined to be those who have injected in the last 6 months, and are categorised by the duration of their current injecting period (< 1 yr/≥ 1 yr) due to the association between duration of injecting period and temporary cessation [24]; those who have been injecting longer are less likely to temporarily cease injecting. When temporarily ceased, PWID may leave the model by permanently ceasing injecting. We assume PWID on OAT are more likely to temporarily cease injecting [24], unless they inject cocaine. The permanent cessation rate and the rates between currently injecting and temporarily ceased injecting are calibrated to data from the study by Xia et al. [24] using submodels. Further details are provided in Section 1.2.

Once infected with HIV, PWID progress through the stages of HIV (acute/latent/pre-AIDS/AIDS) at rates inversely proportional to the average duration of the respective stage of infection. These rates are also dependent on treatment status; those on ART experience slower infection progression and reduced mortality due to AIDS. PWID are infected with HIV at a rate dependent on the number of PWID in each infection stage, the proportion of infected PWID accessing ART, the proportions of PWID with risk factors (homelessness/cocaine injecting), and the proportion accessing OAT. PWID in the acute and pre-AIDS stages of infection transmit at a higher rate. We assume that PWID in the AIDS stage of infection are too sick to mix, and therefore don't contribute to transmission, unless on ART in which case their transmissibility is assumed to be the same as somebody in the pre-AIDS stage on ART. The efficacy of ART at reducing HIV transmission depends on the proportion of PWID on ART who are virally suppressed, which is assumed to increase over the course of the outbreak [17]. A combination of random and assortative mixing based on homelessness, cocaine injecting and geography is assumed.

PWID can either be tested for HIV through systematic testing, or contact tracing. We assume a higher systematic testing rate for PWID who have previously been tested for HIV. The systematic testing rates increase over time and are dependent on homelessness and OAT statuses, to account for the targeted testing interventions in homeless services and pharmacies respectively, and geographical region. Contact tracing moves undiagnosed PWID with HIV into the 'diagnosed' category. The per-capita contact tracing rate is proportional to a rate at which PWID with HIV are asked about contacts (people with whom they've shared injecting equipment), the number of contacts with HIV the average PWID with HIV has (adjusted for the success rate of contact tracing), and the proportion of diagnosed PWID with HIV who have not yet been asked about their contacts.

Diagnosed PWID initiate ART at a time dependent rate to reflect the improvements made to the time from HIV diagnosis to ART initiation throughout the outbreak. Attrition from ART is not included in the model due to the high levels of viral suppression among PWID diagnosed during the outbreak [17, 3] and the short time scale being modelled. The same assumption was made by Flountzi et al. [7] when modelling the HIV

outbreak among PWID in Athens, Greece.

Analysis of the HIV outbreak shows that living with HIV is associated with recent homelessness [5, 15]. HIV testing interventions were also targeted through homeless services and pharmacies, which is assumed to affect PWID experiencing homelessness and PWID on OAT respectively. In the model, PWID can move from being 'never' homeless to 'recently homeless', defined as those who have been homeless in the last 6 months. Those who have been 'recently homeless' can move between the 'recently homeless' and 'previously but not recently homeless' compartments. Different rates are used for becoming homeless first and subsequent times to capture patterns in the data relating to the proportions of PWID who have ever been homeless or been homeless in the last 6 months [5]. A similar structure is used for the OAT component, as shown in Figure 1 of the main text, with 'recently on OAT' defined as being on OAT in the last 6 months. The rates for becoming homeless depend on geography.

Data also shows that there is an association between having an HIV positive status and cocaine injecting [5, 15]. In the model, PWID can start injecting cocaine, but cannot leave the 'injecting cocaine' compartment, unless they stop injecting entirely. This is because the time frame where cocaine injecting started increasing in Glasgow (2015-2020) [5] is relatively short. The rate for initiating cocaine injecting varies over time and by geographical region to account for the increase in cocaine injecting over the course of the outbreak, which was particularly marked in GCC.

The model does not account for PWID movement between geography compartments relating to GCC and RoGGC due to the short time scale being modelled.

1.2 Submodels

Submodels are used to estimate the permanent cessation and relapse rates, and to estimate the temporary cessation rates for PWID not on OAT.

The first submodel follows a cohort of PWID who have temporarily ceased injecting at time $t = 0$. The second submodel follows a cohort of PWID who have started/relapsed injecting at $t = 0$. Both submodels are calibrated to probabilities relating to cessation and relapse calculated by Xia et al. [24] using data from the Edinburgh Addiction Cohort, as detailed in Table S1. In the submodels, we use the proportion of PWID on OAT in the Edinburgh Addiction Cohort, which is 70%, rather than the proportion on OAT from Glasgow.

Schematics are illustrated in Figure S1.

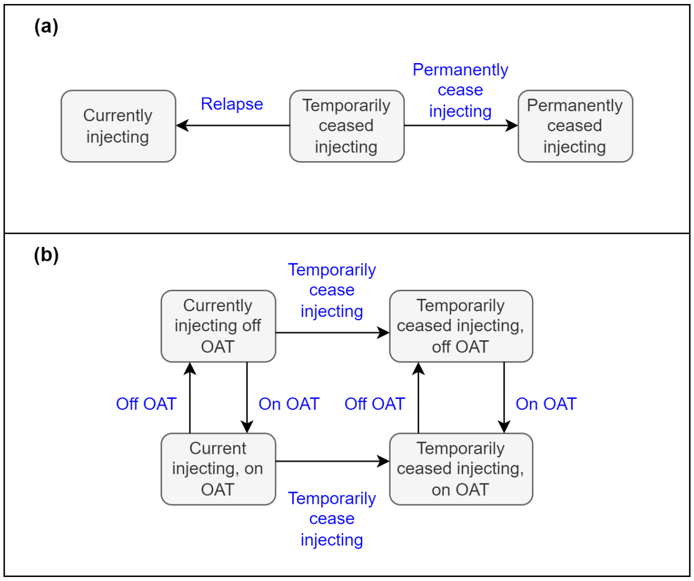

Figure S1: Submodel schematics; (a) to determine the permanent cessation and relapse rates, (b) to determine the permanent cessation and relapse rates.

2 Model equations

Now we'll present the model equations, which provide full detail on the model structure. Information on data used to parameterise the model is provided in Table S1, and information on data used to calibrate the model is provided in Table S2. Unless otherwise specified, all rates refer to per capita rates per year.

2.1 Preliminaries

2.1.1 Defining variables

Let $Y^{i,j,k}_{p,q,r,s}$ be the number of PWID in each compartment where:

- $i \in \mathcal{D}_i = \{1, 2, 3\}$ refers to injecting status (1=currently injecting (<1 yr), 2=currently injecting (≥ 1 yr), 3=temporarily ceased),
- $j \in \mathcal{D}_j = \{1, 2, 3, 4, 5\}$ refers to infection stage (1=susceptible, 2=acute, 3=latent, 4=pre-AIDS, 5=AIDS),
- $k \in \mathcal{D}_k = \{1, 2, 3, 4\}$ refers to diagnosis/treatment status (1=undiagnosed or susceptible and never tested, 2=undiagnosed or susceptible and ever tested, 3=diagnosed and not started ART, 4=diagnosed and started ART),
- $p \in \mathcal{D}_p = \{1, 2, 3\}$ refers to homelessness status (1=never homeless, 2=previously but not recently homeless, 3=recently homeless),
- $q \in \mathcal{D}_q = \{1, 2\}$ refers to cocaine injecting status (1=not injecting cocaine, 2=injecting cocaine),
- $r \in \mathcal{D}_r = \{1, 2, 3\}$ refers to OAT status (1=has never accessed OAT, 2=previously but not recently on OAT, 3=recently on OAT),
- $s \in \mathcal{D}_s = \{1, 2\}$ refers to geographical region (1=GCC, 2=RoGGC).

The model is described by the ODE given by

$$\frac{\mathrm{d}Y^{i,j,k}_{p,q,r,s}}{\mathrm{d}t} = M^{i,j,k}_{p,q,r,s} + \Theta^{i,j,k}_{p,q,r,s} + I^{i,j,k}_{p,q,r,s} + J^{i,j,k}_{p,q,r,s} + K^{i,j,k}_{p,q,r,s} + P^{i,j,k}_{p,q,r,s} + Q^{i,j,k}_{p,q,r,s} + R^{i,j,k}_{p,q,r,s} \tag{1}$$

where

- $M^{i,j,k}_{p,q,r,s}$ describes non-AIDS related mortality and permanent cessation of injecting,
- $\Theta^{i,j,k}_{p,q,r,s}$ describes recruitment into the model,
- $I^{i,j,k}_{p,q,r,s}$ describes movement between current and temporary cessation of drug use,
- $J^{i,j,k}_{p,q,r,s}$ describes HIV transmission and infection progression,
- $K^{i,j,k}_{p,q,r,s}$ describes testing and treatment for HIV,
- $P^{i,j,k}_{p,q,r,s}$ describes movement between homelessness categories,
- $Q^{i,j,k}_{p,q,r,s}$ describes movement between cocaine injecting categories,
- $R^{i,j,k}_{p,q,r,s}$ describes movement between OAT categories.

2.1.2 Notation

Sums over multiple indices are expressed using a single summation symbol with all indices being summed over in the subscript. Unless otherwise stated, the domain being summed over is all possible values of the index, as outlined above. For example,

$$\sum_{i,j,k,p,q,r,s} Y^{i,j,k}_{p,q,r,s} = \sum_{i\in\mathcal{D}_i}\sum_{j\in\mathcal{D}_j}\sum_{k\in\mathcal{D}_k}\sum_{p\in\mathcal{D}_p}\sum_{q\in\mathcal{D}_q}\sum_{r\in\mathcal{D}_r}\sum_{s\in\mathcal{D}_s} Y^{i,j,k}_{p,q,r,s}. \tag{2}$$

The notation $[i..i+n]$ is used to refer to the set of consecutive integers from i to $i+n$ inclusive, $\{i, i+1, i+2, ..., i+n-1, i+n\}$.

The population sizes $Y^{i,j,k}_{p,q,r,s}$ and the rates outlined in Section 2.1.1 are dependent on time, but the time dependence is only explicitly written where there is ambiguity.

2.2 Leaving the model

PWID leave the model through death or permanent cessation of injecting drug use. AIDS mortality is accounted for in Section 2.5; the other death rates in the model are:

- ω^{nDRD}: background mortality rate,
- $\omega^{DRD}(t)$: drug related mortality rate.

The drug related death rate $\omega^{DRD}(t)$ is time dependent;

$$\omega^{DRD}(t) = \begin{cases} \omega^{DRD}_{2013} & \text{if } t < 2013.5, \\ \omega^{DRD}_{2013} + \left(\frac{t-2013.5}{2018.5-2013.5}\right)\left(\omega^{DRD}_{2018} - \omega^{DRD}_{2013}\right) & \text{if } 2013.5 \leq t < 2018.5, \\ \omega^{DRD}_{2018} & \text{if } t \geq 2018.5, \end{cases} \tag{3}$$

where ω^{DRD}_{2013} and ω^{DRD}_{2018} are the drug related death rates in 2013 and 2018 respectively. Note that the time dependent function has been set up so that these rates are fixed in the middle of the year they refer to, rather than the beginning.

Let η be the rate for permanent cessation of injecting drug use.

$M^{i,j,k}_{p,q,r,s}$ is the component of the ODE which describes how PWID leave the model for non-AIDS related reasons and can be expressed as

$$\begin{aligned} M^{1,j,k}_{p,q,r,s} &= -(\omega^{nDRD} + \omega^{DRD}(t))Y^{1,j,k}_{p,q,r,s}, \\ M^{2,j,k}_{p,q,r,s} &= -(\omega^{nDRD} + \omega^{DRD}(t))Y^{2,j,k}_{p,q,r,s}, \\ M^{3,j,k}_{p,q,r,s} &= -\left(\omega^{nDRD} + \eta\right)Y^{3,j,k}_{p,q,r,s}. \end{aligned} \tag{4}$$

2.3 Recruitment

PWID enter the model as currently injecting, susceptible to HIV, not tested for HIV and not recently on OAT. They may enter into the model with various homelessness, cocaine injecting and geographical statuses according to:

- $p^H_{p,s}$: the proportion entering in each homelessness category (p) according to geography status (s),
- $p^C_{q,s}(t)$: the proportion entering in each cocaine injecting category (q) according to geography status (s),
- p^G_s: the proportion entering in each geography category (s).

The proportion entering as injecting cocaine $p^C_{q,s}(t)$ depends on time;

$$p^C_{q,s}(t) = \begin{cases} \tilde{p}^{pre/early}_{q,s} & \text{if } t < 2016, \\ \tilde{p}^{pre/early}_{q,s} + \left(\frac{t-2016}{2018-2016}\right)\left[\tilde{p}^{mid/late}_{q,s} - \tilde{p}^{pre/early}_{q,s}\right] & \text{if } 2016 \leq t < 2018, \\ \tilde{p}^{mid/late}_{q,s} & \text{if } t \geq 2018. \end{cases} \tag{5}$$

where $\tilde{p}^{pre/early}$ and $\tilde{p}^{mid/late}$ refer to the average proportions in the pre/early outbreak eras (pre-2017) and the mid/late outbreak eras (2017 onwards) respectively. A smooth transition from the pre/early rate to the mid/late rate has been implemented to avoid discrete jumps in the model output.

The recruitment rate into the model is set to balance those exiting the model due to non-AIDS related mortality in 2013 (before the increase in drug related deaths) and permanent cessation of injecting drug use.

The overall recruitment rate, θ, can be written as

$$\theta = \left(\omega^{nDRD} + \omega^{DRD}(2013)\right) \sum_{i\in\{1,2\},j,k,p,q,r,s} Y^{i,j,k}_{p,q,r,s} + \left(\omega^{nDRD} + \eta\right) \sum_{j,k,p,q,r,s} Y^{3,j,k}_{p,q,r,s}, \tag{6}$$

where ω^{nDRD}, $\omega^{DRD}(t)$ and η are the background mortality, drug related mortality and permanent cessation rates respectively, defined in Section 2.2.

It follows that

$$\begin{aligned} \Theta^{1,1,1}_{p,q,1,s} &= p^H_{p,s} p^C_{q,s} p^G_s \theta, \\ \Theta^{i,j,k}_{p,q,r,s} &= 0 \text{ for all other values of } i,j,k,p,q,r,s. \end{aligned} \tag{7}$$

2.4 Injecting status

PWID move between current and temporarily ceased injecting compartments according to:

- κ^{inj}: the rate at which temporarily ceased PWID relapse back to injecting,
- $\kappa^{(c1)}$: the rate at which currently injecting (<1 yr) PWID temporarily cease injecting,
- $\kappa^{(c2)}$: the rate at which currently injecting (≥ 1 yr) PWID temporarily cease injecting.

These rates are affected by:

- F^{cease}_r: the effect of OAT status (r) on the rate of temporary cessation of injecting - note that $F^{cease}_1 = F^{cease}_2 = 1$. This only applies to PWID not injecting cocaine.

The injecting status component of the model, $I^{i,j,k}_{p,q,r,s}$, can be expressed as

$$\begin{aligned} I^{1,j,k}_{p,1,r,s} &= \kappa^{inj} Y^{3,j,k}_{p,1,r,s} - F^{cease}_r \kappa^{(c1)} Y^{1,j,k}_{p,1,r,s} - Y^{1,j,k}_{p,1,r,s}, \\ I^{2,j,k}_{p,1,r,s} &= -F^{cease}_r \kappa^{(c2)} Y^{2,j,k}_{p,1,r,s} + Y^{1,j,k}_{p,1,r,s}, \\ I^{3,j,k}_{p,1,r,s} &= F^{cease}_r \kappa^{(c1)} Y^{1,j,k}_{p,1,r,s} + F^{cease}_r \kappa^{(c2)} Y^{2,j,k}_{p,1,r,s} - \kappa^{inj} Y^{3,j,k}_{p,1,r,s}, \\ I^{1,j,k}_{p,2,r,s} &= \kappa^{inj} Y^{3,j,k}_{p,2,r,s} - \kappa^{(c1)} Y^{1,j,k}_{p,2,r,s} - Y^{1,j,k}_{p,2,r,s}, \\ I^{2,j,k}_{p,2,r,s} &= -\kappa^{(c2)} Y^{2,j,k}_{p,2,r,s} + Y^{1,j,k}_{p,2,r,s}, \\ I^{3,j,k}_{p,2,r,s} &= \kappa^{(c1)} Y^{1,j,k}_{p,2,r,s} + \kappa^{(c2)} Y^{2,j,k}_{p,2,r,s} - \kappa^{inj} Y^{3,j,k}_{p,2,r,s}. \end{aligned} \tag{8}$$

2.5 Infection stage/transmission

Let $\Lambda^i_{p,q,r,s}$ be the force of infection, which depends on the injecting status (i), homelessness status (p), cocaine injecting status (q), OAT status (r) and geographical region (s) of the individual being infected. It also depends on these attributes of the infected person, as well as their ART status. It is assumed those who are not currently injecting cannot acquire the virus or transmit the virus. Homelessness, cocaine injecting and ART statuses are included within the force of infection because they affect the transmission dynamics. Homelessness, cocaine injecting and geography account for some degree of assortative mixing.

2.5.1 Infectors

First, let's consider the transmissibility of individuals with HIV. This depends on:

- $\psi^{(1)}$: the transmissibility in the acute stage of infection,
- $\psi^{(2)}$ the transmissibility in the latent stage of infection,
- $\psi^{(3)}$, the transmissibility in the pre-AIDS stage of infection,
- T^{inf} the effect of ART on transmission.

We consider PWID on AIDS to be too sick to mix unless they are on ART, in which case they have they same infectivity as an individual on ART in the pre-AIDS stage.

The effect of ART on transmission, T^{inf}, is constructed from data related to the plasma viral load (PVL) and viral suppression:

- $\phi(t)$: the probability that a PWID with HIV on ART is virally suppressed - this is time-dependent to reflect the improvements in adherence to ART treatment which occurred as a result of Glasgow Enhanced Care HIV outreach GECHO intervention [17],
- Δ^S: the log (base 10) difference between the PVL at the start of ART (copies/ml) and the PVL after 1.5 yrs for those virally suppressed (copies/ml),
- Δ^U: the log (base 10) difference between the PVL at the start of ART (copies/ml) and the PVL after 1.5 yrs for those not virally suppressed (copies/ml),
- υ: the factor describing the increase in HIV transmissibility per $\log_{10}$ increase in PVL.

The proportion of PWID with HIV on ART who are virally suppressed can be expressed as:

$$\phi(t) = \begin{cases} \phi^{pre} & \text{if } t < 2016.5, \\ \phi^{pre} + \frac{t-2016.5}{2018.5-2016.5}\left(\phi^{post} - \phi^{pre}\right) & \text{if } 2016.5 \leq t < 2018.5, \\ \phi^{post} & \text{if } t \geq 2018.5, \end{cases} \tag{9}$$

where ϕ^{pre} and ϕ^{post} refer to the proportions who are virally suppressed before and after improvements in ART retention are observed respectively. The data used to parametrise these values refer to the average proportion virally suppressed before mid-2016 and after mid-2018 respectively.

The relative decrease in HIV transmission risk for PWID on ART who are virally suppressed and unsuppressed can be expressed as

$$\begin{aligned} \delta_s &= \frac{1}{\upsilon^{\Delta^S}}, \\ \delta_u &= \frac{1}{\upsilon^{\Delta^U}}, \end{aligned} \tag{10}$$

respectively.

This gives the average prevention effectiveness of ART to be

$$T^{inf} = \phi(t)\delta_s + (1 - \phi(t))\delta_u. \tag{11}$$

Now we can construct a variable $X_{p,q,r,s}$ which accounts for the number of infectious PWID in each homelessness (p), cocaine injecting (q), OAT (r) and geography (s) category, weighted to account for the effect of infection stage and ART status on infectivity:

$$\begin{aligned} X_{p,q,r,s} =& \frac{\psi^{(1)}}{\psi^{(2)}} \sum_{i\in\{1,2\},k\in[1..3]} Y^{i,2,k}_{p,q,r,s} + \sum_{i\in\{1,2\},k\in[1..3]} Y^{i,3,k}_{p,q,r,s} + \frac{\psi^{(3)}}{\psi^{(2)}} \sum_{i\in\{1,2\},k\in[1..3]} Y^{i,4,k}_{p,q,r,s} \\ &+ T^{inf}\left[\frac{\psi^{(1)}}{\psi^{(2)}} \sum_{i\in\{1,2\}} Y^{i,2,4}_{p,q,r,s} + \sum_{i\in\{1,2\}} Y^{i,3,4}_{p,q,r,s} + \frac{\psi^{(3)}}{\psi^{(2)}} \sum_{i\in\{1,2\}} Y^{i,4,4}_{p,q,r,s} + \frac{\psi^{(3)}}{\psi^{(2)}} \sum_{i\in\{1,2\}} Y^{i,5,4}_{p,q,r,s}\right]. \end{aligned} \tag{12}$$

2.5.2 Mixing

The degree to which someone mixes (i.e. the probability of a contact which could result in transmission) depends on:

- σ^H: the proportion of assortative mixing according to homelessness status,
- σ^C: the proportion of assortative mixing according to cocaine injecting status,
- σ^G: the proportion of assortative mixing according to geography status,
- H^{inf}_p: the effect of homelessness status (p) on mixing - note that $H^{inf}_1 = H^{inf}_2 = 1$,
- C^{inf}_q: the effect of cocaine injecting status (q) on mixing - note that $C^{inf}_1 = 1$,
- F^{inf}_r: the effect of OAT status (r) on mixing - note that $F^{inf}_1 = F^{inf}_2 = 1$.

We use these parameters to define some further intermediary variables $V^{\{label\}}$ which describe the probability of contact with an infected individual, adjusted for the effects of homelessness, cocaine injecting and OAT. The superscript refers to the type of mixing; $rand$ = random, H = by homelessness status, C = by cocaine injecting status, HC = by both cocaine injecting and homelessness statuses, G = by geography status, HG = by homelessness and geography statuses, CG = by cocaine injecting and geography statuses, HCG = by homelessness, cocaine injecting and geography statuses.

$$V^{rand} = \frac{\sum\limits_{p,q,r,s} H_p^{inf} C_q^{inf} F_r^{inf} X_{p,q,r,s}}{\sum\limits_{\substack{i\in\{1,2\},j\in[1..4],k,\\ p,q,r,s}} H_p^{inf} C_q^{inf} F_r^{inf} Y_{p,q,r,s}^{i,j,k} + \sum\limits_{i\in\{1,2\},p,q,r,s} H_p^{inf} C_q^{inf} F_r^{inf} Y_{p,q,r,s}^{i,5,4}}, \tag{13}$$

$$V_p^{H} = \frac{\sum\limits_{q,r,s} C_q^{inf} F_r^{inf} X_{p,q,r,s}}{\sum\limits_{\substack{i\in\{1,2\},j\in[1..4],k,\\ q,r,s}} C_q^{inf} F_r^{inf} Y_{p,q,r,s}^{i,j,k} + \sum\limits_{i\in\{1,2\},q,r,s} C_q^{inf} F_r^{inf} Y_{p,q,r,s}^{i,5,4}}, \tag{14}$$

$$V_q^{C} = \frac{\sum\limits_{p,r,s} H_p^{inf} F_r^{inf} X_{p,q,r,s}}{\sum\limits_{\substack{i\in\{1,2\},j\in[1..4],k,\\ p,r,s}} H_p^{inf} F_r^{inf} Y_{p,q,r,s}^{i,j,k} + \sum\limits_{i\in\{1,2\},p,r,s} H_p^{inf} F_r^{inf} Y_{p,q,r,s}^{i,5,4}}, \tag{15}$$

$$V_{p,q}^{HC} = \frac{\sum\limits_{r,s} F_r^{inf} X_{p,q,r,s}}{\sum\limits_{\substack{i\in\{1,2\},j\in[1..4],k,\\ r,s}} F_r^{inf} Y_{p,q,r,s}^{i,j,k} + \sum\limits_{i\in\{1,2\},r,s} F_r^{inf} Y_{p,q,r,s}^{i,5,4}}, \tag{16}$$

$$V_s^{G} = \frac{\sum\limits_{p,q,r} H_p^{inf} C_q^{inf} F_r^{inf} X_{p,q,r,s}}{\sum\limits_{\substack{i\in\{1,2\},j\in[1..4],k,\\ p,q,r}} H_p^{inf} C_q^{inf} F_r^{inf} Y_{p,q,r,s}^{i,j,k} + \sum\limits_{i\in\{1,2\},p,q,r} H_p^{inf} C_q^{inf} F_r^{inf} Y_{p,q,r,s}^{i,5,4}}, \tag{17}$$

$$V_{p,s}^{HG} = \frac{\sum\limits_{q,r} C_q^{inf} F_r^{inf} X_{p,q,r,s}}{\sum\limits_{\substack{i\in\{1,2\},j\in[1..4],k,\\ q,r}} C_q^{inf} F_r^{inf} Y_{p,q,r,s}^{i,j,k} + \sum\limits_{i\in\{1,2\},q,r} C_q^{inf} F_r^{inf} Y_{p,q,r,s}^{i,5,4}}, \tag{18}$$

$$V_{q,s}^{CG} = \frac{\sum\limits_{p,r} H_p^{inf} F_r^{inf} X_{p,q,r,s}}{\sum\limits_{\substack{i\in\{1,2\},j\in[1..4],k,\\ p,r}} H_p^{inf} F_r^{inf} Y_{p,q,r,s}^{i,j,k} + \sum\limits_{i\in\{1,2\},p,r} H_p^{inf} F_r^{inf} Y_{p,q,r,s}^{i,5,4}}, \tag{19}$$

$$V_{p,q,s}^{HCG} = \frac{\sum\limits_{r} F_r^{inf} X_{p,q,r,s}}{\sum\limits_{\substack{i\in\{1,2\},j\in[1..4],k,\\ r}} F_r^{inf} Y_{p,q,r,s}^{i,j,k} + \sum\limits_{i\in\{1,2\},r} F_r^{inf} Y_{p,q,r,s}^{i,5,4}}. \tag{20}$$

2.5.3 Force of infection

We can now construct the force of infection $\Lambda^{i}_{p,q,r,s}$, taking mixing into account:

$$
\begin{aligned}
\Lambda^{1}_{p,q,r,s} = \lambda H^{inf}_{p} C^{inf}_{q} F^{inf}_{r} \Bigg[& \sigma^{H} \Big(\sigma^{C} \Big(\sigma^{G} V^{HCG}_{p,q,s} + (1-\sigma^{G}) V^{HC}_{p,q} \Big) \\
& + (1-\sigma^{C}) \Big(\sigma^{G} V^{HG}_{p,s} + (1-\sigma^{G}) V^{H}_{p} \Big) \Big) \\
& + (1-\sigma^{H}) \Big(\sigma^{C} \Big(\sigma^{G} V^{CG}_{q,s} + (1-\sigma^{G}) V^{C}_{q} \Big) \\
& + (1-\sigma^{C}) \Big(\sigma^{G} V^{G}_{s} + (1-\sigma^{G}) V^{rand} \Big) \Big) \Bigg], \\
\Lambda^{2}_{p,q,r,s} = \Lambda^{1}_{p,q,r,s}&, \\
\Lambda^{3}_{p,q,r,s} = 0&,
\end{aligned} \tag{21}
$$

where λ refers to the per-capita infection rate.

Note that the homelessness, cocaine injecting and OAT categories affect both the infector and susceptible individuals in a transmission interaction. The relative risk factors appear in the V variables (Equations 13 - 20) to reflect the effect of homelessness, cocaine injecting and OAT on the volume of mixing of an infector. The terms appear in $\Lambda^{i}_{p,q,r,s}$ (Equation 21) to reflect the effect of homelessness, cocaine injecting and OAT on the volume of mixing of a susceptible individual.

2.5.4 Infection Progression

HIV progression depends on:

- $\tau^{(1)}$: the rate of infection progression in the acute stage,
- $\tau^{(2)}$: the rate of infection progression in the latent stage,
- $\tau^{(3)}$: the rate of infection progression in the pre-AIDS stage,
- $\tau^{(4)}$: the AIDS-related mortality rate,
- T^{prog}_{k}: the effect of ART status (k) on infection progression - note that $T^{prog}_{k} = 1$ for $k = [1..3]$.

The infection progression rates are calculated using the average durations in each infection stage:

- $\tau^{(1)} = \frac{12}{d^{(1)}}$, where $d^{(1)}$ is the duration of the acute stage in months,
- $\tau^{(3)} = \frac{12}{d^{(3)}}$, where $d^{(3)}$ is the duration of the pre-AIDS stage in months,
- $\tau^{(4)} = \frac{12}{d^{(4)}}$, where $d^{(4)}$ is the duration of the AIDS stage in months,
- $\tau^{(2)} = \frac{12}{12d^{(0)} - d^{(1)} - d^{(3)}}$, where $d^{(0)}$ is the duration from infection to AIDS in years, so that the denominator is the duration of the latent stage in months.

Using these parameters and the force of infection $\Lambda^{i}_{p,q,r,s}$ defined above, the infection progression component of the model $J^{i,j,k}_{p,q,r,s}$ can be constructed as follows:

$$
\begin{aligned}
J^{i,1,k}_{p,q,r,s} &= -\Lambda^{i}_{p,q,r,s} Y^{i,1,k}_{p,q,r,s}, \\
J^{i,2,k}_{p,q,r,s} &= \Lambda^{i}_{p,q,r,s} Y^{i,1,k}_{p,q,r,s} - T^{prog}_{k} \tau^{(1)} Y^{i,2,k}_{p,q,r,s}, \\
J^{i,3,k}_{p,q,r,s} &= T^{prog}_{k} \left(\tau^{(1)} Y^{i,2,k}_{p,q,r,s} - \tau^{(2)} Y^{i,3,k}_{p,q,r,s} \right), \\
J^{i,4,k}_{p,q,r,s} &= T^{prog}_{k} \left(\tau^{(2)} Y^{i,3,k}_{p,q,r,s} - \tau^{(3)} Y^{i,4,k}_{p,q,r,s} \right), \\
J^{i,5,k}_{p,q,r,s} &= T^{prog}_{k} \left(\tau^{(3)} Y^{i,4,k}_{p,q,r,s} - \tau^{(4)} Y^{i,5,k}_{p,q,r,s} \right).
\end{aligned} \tag{22}
$$

2.6 Testing and treatment for HIV

2.6.1 Setting up testing rates

There are two categories of HIV testing in the model; systematic testing (a population level intervention) and contact tracing (an individual level intervention). In the model, separate testing rates are used for HIV tests carried out in drug services and other locations. The parameters in the model related to systematic testing are:

- $\alpha^{GCC}(t)$: a time-dependent function for the first testing rate in drug services for PWID who are not homeless, not on OAT and who live in GCC,
- $\alpha^{RoGGC}(t)$: a time-dependent function for the first testing rate in drug services for PWID who are not homeless, not on OAT and who live in RoGGC,
- $\beta^{GCC}(t)$: a time-dependent function for the first testing rate in other locations for PWID who live in GCC,
- $\beta^{RoGGC}(t)$: a time-dependent function for the first testing rate in other locations for PWID who live in RoGGC,
- $H_p^{test}(t)$: a time-dependent function for the effect of homelessness status (p) on testing rates in drug services - note that $H_1^{test}(t) = H_2^{test}(t) = 1$,
- $F_r^{test}(t)$: a time dependent function for the effect of OAT status (r) on testing rates in drug services - note that $F_1^{test}(t) = F_2^{test}(t) = 1$,
- R^{repeat}: the ratio of repeat to first testing rates.

The effect of homelessness and OAT on testing are assumed to be time-dependent to reflect targeted testing strategies which were implemented as part of the outbreak [23]. We assume that it takes one year for the interventions to have maximum effect.

Interventions which aimed to improve HIV testing rates amongst people experiencing homelessness began June/July 2016:

$$H_3^{test}(t) = \begin{cases} h_{pre}^{test} & \text{if } t < 2016.5, \\ h_{pre}^{test} + \left(\frac{t-2016.5}{2017.5-2016.5}\right) h_{post}^{test} - h_{pre}^{test}] & \text{if } 2016.5 \leq t < 2017.5, \\ h_{post}^{test} & \text{if } t \geq 2017.5, \end{cases} \tag{23}$$

where h_{pre}^{test} and h_{post}^{test} are the effects of homelessness on testing in the 'pre' and 'post' intervention eras respectively. The effect 'pre' intervention, h_{pre}^{test}, is set to 1 in the model, as outlined in Table S1.

Increased testing for PWID on OAT began in May 2015:

$$F_3^{test}(t) = \begin{cases} d_{pre}^{test} & \text{if } t < 2015.5, \\ d_{pre}^{test} + \left(\frac{t-2015.5}{2016.5-2015.5}\right) d_{post}^{test} - d_{pre}^{test}] & \text{if } 2015.5 \leq t < 2016.5, \\ d_{post}^{test} & \text{if } t \geq 2016.5, \end{cases} \tag{24}$$

where d_{pre}^{test} and d_{post}^{test} are the effects of OAT on testing in the 'pre' and 'post' intervention eras respectively.

As a function of time, the first testing rate in drug services for PWID who are not homeless, not on OAT and who live in GCC is

$$\alpha^{GCC}(t) = \begin{cases} \alpha_{pre}^{GCC} & \text{if } t < 2015, \\ \alpha_{pre}^{GCC} + \left(\frac{t-2015}{2016-2015}\right)\tilde{\alpha}_{early}^{GCC} & \text{if } 2015 \leq t < 2016, \\ \alpha_{pre}^{GCC} + \tilde{\alpha}_{early}^{GCC} + \left(\frac{t-2016}{2018-2016}\right)\tilde{\alpha}_{mid}^{GCC} & \text{if } 2016 \leq t < 2018, \\ \alpha_{pre}^{GCC} + \tilde{\alpha}_{early}^{GCC} + \tilde{\alpha}_{mid}^{GCC} + \left(\frac{t-2018}{2020-2018}\right)\tilde{\alpha}_{late}^{GCC} & \text{if } 2018 \leq t < 2020, \\ \alpha_{pre}^{GCC} + \tilde{\alpha}_{early}^{GCC} + \tilde{\alpha}_{mid}^{GCC} + \tilde{\alpha}_{late}^{GCC} & \text{if } t \geq 2020, \end{cases} \tag{25}$$

where α_{pre}^{GCC} is the first testing rate in GCC in the pre-outbreak era (2013-2014), and $\tilde{\alpha}_{early}^{GCC}$, $\tilde{\alpha}_{mid}^{GCC}$, $\tilde{\alpha}_{late}^{GCC}$ refer to the absolute increases in the first testing rate in the early- (2015-2016), mid- (2017-2018) and late- (2019-2020) outbreak eras respectively. The time points for the changes coincide with the mid-points of these eras, except for the first change because we know the outbreak was detected in 2015. The data used to inform these rates comes from the biennial NESI survey, which shows the rate of scale-up is different between GCC and RoGGC. Considering the testing rates over the four periods of the outbreak allows this to be incorporated into the model.

The same structure is be used for tests in other locations, with the time dependence adjusted to give a shallower gradient. We parameterise this using the proportion of HIV tests carried out in drug services in 2020, w_s^{OL}, which depends on geography status (s). To get expressions for the testing rates in other locations, we first construct an expression for the ratio of the testing rates in other locations to the rates in drug services, r_s^{OL}, which also depends on geography status (s).

The total number of tests carried out in drug services in 2020 in GGC is

$$\sum_{\substack{i,j,k,\\p,q,r}} \int_{2020}^{2021} H_p^{test}(t')F_r^{test}(t')\left(1 + R^{repeat}\right)\alpha^{GCC}(t')Y_{p,q,r,1}^{i,j,k}(t')\,\mathrm{d}t'\,. \tag{26}$$

which can be approximated by

$$\sum_{\substack{i,j,k,\\p,q,r}} H_p^{test}(2020)F_r^{test}(2020)\left(1 + R^{repeat}\right)\alpha^{GCC}(2020)Y_{p,q,r,1}^{i,j,k}(t_0) \tag{27}$$

if we assume the numbers in each category are approximately constant over the course of 2020 and are well represented by the initial conditions of the system at time $t = t_0$. Note that $H_p^{test}(t)$, $F_r^{test}(t)$ and $\alpha^{GCC}(t)$ are constant for $t \in [2020, 2021]$.

Similarly, the total number of tests carried out in other locations in 2020 in GGC can be approximated by

$$\sum_{\substack{i,j,k,\\p,q,r}} r_1^{OL}\left(1 + R^{repeat}\right)\alpha^{GCC}(2020)Y_{p,q,r,1}^{i,j,k}(t_0) \tag{28}$$

where r_1^{OL} is the ratio for the testing rate in other locations compared to the rate in drug services in GCC. Note that homelessness and OAT statuses don't affect the number of tests carried out in other locations.

The proportion of tests carried out in drug services can be expressed in terms of the number of HIV tests carried out in drugs services and other locations,

$$
\begin{aligned}
w_1^{OL} &= \frac{\text{No. tests in drug services}}{\text{No. tests in drug services} + \text{No. tests in other locations}} \\
&\approx \frac{1}{1 + \left(\frac{\sum_{\substack{i,j,k,\\p,q,r}} r_1^{OL}(1+R^{repeat})\alpha^{GCC}(2020)Y_{p,q,r,1}^{i,j,k}(t_0)}{\sum_{\substack{i,j,k,\\p,q,r}} H_p^{test}(2020)F_r^{test}(2020)(1+R^{repeat})\alpha^{GCC}(2020)Y_{p,q,r,1}^{i,j,k}(t_0)} \right)}.
\end{aligned}
\tag{29}
$$

We can rearrange this to get an expression for the ratio for the testing rate in other locations to the rate in drug services,

$$
r_1^{OL} = \left(\frac{1 - w_1^{OL}}{w_1^{OL}} \right) \frac{H_p^{test}(2020)F_r^{test}(2020)Y_{p,q,r,1}^{i,j,k}(t_0)}{\sum_{\substack{i,j,k,\\p,q,r}} Y_{p,q,r,1}^{i,j,k}(t_0)}. \tag{30}
$$

The ratio for the testing rate in other locations compared to the rate in drug services in RoGGC, r_2^{OL}, can be constructed in a similar way.

Before defining the testing rate from other locations, we will define some intermediary variables which represent the testing rate in the pre-outbreak era (β_{pre}^{GCC}) and the absolute increases in the early-, mid- and late-outbreak eras ($\tilde{\beta}_{early}^{GCC}$, $\tilde{\beta}_{mid}^{GCC}$ and $\tilde{\beta}_{late}^{GCC}$ respectively),

$$
\begin{aligned}
\beta_{pre}^{GCC} &= r^{OL} \left(\alpha^{GCC} + (1-q) \left(\tilde{\alpha}_{early}^{GCC} + \tilde{\alpha}_{mid}^{GCC} + \tilde{\alpha}_{late}^{GCC} \right) \right), \\
\tilde{\beta}_{early}^{GCC} &= r^{OL} q \tilde{\alpha}_{early}^{GCC}, \\
\tilde{\beta}_{mid}^{GCC} &= r^{OL} q \tilde{\alpha}_{mid}^{GCC}, \\
\tilde{\beta}_{late}^{GCC} &= r^{OL} q \tilde{\alpha}_{late}^{GCC},
\end{aligned}
\tag{31}
$$

where q is the time dependence proportion relative to tests in drug services. So if $q = 0.5$, then the gradient over time of the testing rate in other locations is half the gradient over time of tests in drug services.

Then the testing rate in other locations can be expressed as,

$$
\beta^{GCC}(t) = \begin{cases}
\beta_{pre}^{GCC} & \text{if } t < 2015, \\
\beta_{pre}^{GCC} + \left(\frac{t-2015}{2016-2015} \right) \tilde{\beta}_{early}^{GCC} & \text{if } 2015 \leq t < 2016, \\
\beta_{pre}^{GCC} + \tilde{\beta}_{early}^{GCC} + \left(\frac{t-2016}{2018-2016} \right) \tilde{\beta}_{mid}^{GCC} & \text{if } 2016 \leq t < 2018, \\
\beta_{pre}^{GCC} + \tilde{\beta}_{early}^{GCC} + \tilde{\beta}_{mid}^{GCC} + \left(\frac{t-2018}{2020-2018} \right) \tilde{\beta}_{late}^{GCC} & \text{if } 2018 \leq t < 2020, \\
\beta_{pre}^{GCC} + \tilde{\beta}_{early}^{GCC} + \tilde{\beta}_{mid}^{GCC} + \tilde{\beta}_{late}^{GCC} & \text{if } t \geq 2020.
\end{cases}
\tag{32}
$$

The corresponding testing rates in drug services and other locations for RoGGC, α^{RoGGC} and β^{RoGGC} respectively, are constructed in a similar way.

2.6.2 Contact tracing

The parameters relevant to contact tracing are:

- the contact tracing rate, $\epsilon(t)$,
- the average number of contacts with HIV from one person with HIV, adjusted for contact tracing success, χ.

The contact tracing rate, $\epsilon(t)$, is the rate at which people who have been diagnosed are asked about their contacts. This has been included in the model as a time dependent function,

$$\epsilon(t) = \begin{cases} 0 & \text{if } t < 2015.25, \\ \left(\frac{t-2015.25}{2015.5-2015.25}\right)\epsilon & \text{if } 2015.25 \leq t < 2015.5, \\ \\ \epsilon & \text{if } t \geq 2015.5, \end{cases} \tag{33}$$

where ϵ is the final contact tracing rate. We assume that contact tracing was initiated at the beginning of the second quarter in 2015, and reached full capacity mid-way through 2015. A smooth transition was incorporated to avoid discrete jumps in the parameters of the model.

An approach similar to Sturniolo et al. [21] has been used, where the per-capita rate at which people in a group Y_G are identified through contact tracing is given by

$$\epsilon(t)\chi \Pr(C_I|Y_G), \tag{34}$$

where the term $\Pr(C_I|Y_G)$ is the probability of being a contact of someone who is infected (C_I) conditional on membership of group Y_G.

Here we only apply contact tracing in the model to individuals with HIV. Susceptible people aren't isolated due to contact tracing for HIV, so the only way contact tracing may affect them is by changing their testing rate. However, we assume that being contact traced does not impact engagement with services. Furthermore, the number of susceptible PWID contact traced is small compared to the overall number of HIV tests so this assumption is likely to have little impact on the model results.

If somebody has HIV, then they must have had contact with an infected individual. We assume that the probability of contact with an infected individual who is being asked about contacts is $\frac{D^{NCT}}{Y_I}$ where D^{NCT} is the total number of people diagnosed with HIV who have not been asked about contact tracing, and $Y_I = \sum_{\substack{i,j \in [2..5],k, \\ p,q,r,s}} Y^{i,j,k}_{p,q,r,s}$ is the total number of people infected with HIV. Therefore, the per-capita contact tracing rate is

$$\epsilon(t)\chi \frac{D^{NCT}}{Y_I}. \tag{35}$$

Note that we haven't accounted for differences in the probability of contact due to injecting status. However, the number of people infected who are not currently injecting is likely to be small compared to the number of people infected who are currently injecting because we assume only those who are currently injecting can acquire HIV.

The equation for the total number of people diagnosed and not asked about contacts can be approximated by

$$
\begin{aligned}
\frac{\mathrm{d}D^{NCT}}{\mathrm{d}t} = &\left(\sum_{\substack{i,j,k\in\{3,4\},\\ p,q,r,s}} M^{i,j,k}_{p,q,r,s} - \sum_{\substack{i,k\in\{3,4\},\\ p,q,r,s}} T^{prog}_k \tau^{(4)} Y^{i,5,k}_{p,q,r,s}\right)\left(\frac{D^{NCT}}{\sum_{\substack{i,j\in[2..5],k\in\{3,4\},\\ p,q,r,s}} Y^{i,j,k}_{p,q,r,s}}\right)\\
&+ \sum_{\substack{i,j\in[2..5],\\ p,q,r}} \Big(H^{test}_p F^{test}_r \Big(\alpha^{GCC} Y^{i,j,1}_{p,q,r,1} + \alpha^{RoGGC} Y^{i,j,1}_{p,q,r,2}\\
&+ R^{repeat}\alpha^{GCC} Y^{i,j,2}_{p,q,r,1} + R^{repeat}\alpha^{RoGGC} Y^{i,j,2}_{p,q,r,2}\Big)\\
&+ \beta^{GCC}\left(Y^{i,j,1}_{p,q,r,1} + R^{repeat} Y^{i,j,2}_{p,q,r,1}\right) + \beta^{RoGGC}\left(Y^{i,j,1}_{p,q,r,2} + R^{repeat} Y^{i,j,2}_{p,q,r,2}\right)\Big)\\
&+ \epsilon(t)\chi\left(\frac{D^{NCT}}{\sum_{\substack{i,j\in[2..5],k,\\ p,q,r,s}} Y^{i,j,k}_{p,q,r,s}}\right) \sum_{\substack{i,j\in[2..5],k\in\{1,2\},\\ p,q,r,s}} Y^{i,j,k}_{p,q,r,s}\\
&- \epsilon(t) D^{NCT}
\end{aligned} \tag{36}
$$

where the first line refers to mortality and permanent cessation of injecting, the second and third lines refer to testing in drug services, the fourth line refers to testing in other locations, the fifth line refers to contact tracing and the final line refers to people being asked about contacts. The approximation is in the death rate. We have assumed that the death rate for people diagnosed and not yet asked about contacts can be approximately by the death rate for people who have been diagnosed multiplied by the proportion of people who are diagnosed who have not yet been asked about contacts. Model complexity would be increased if we didn't make this assumption because we would have to track the numbers diagnosed and not asked about contacts in each of the individual compartments in the model. The simplifying assumption is justified because the per capita mortality and permanent cessation rates are much smaller (by a factor of order 100) than the per capita contact tracing rate. Any differences due to the simplifying assumption are much smaller than the parameter uncertainty in the model.

2.6.3 Treatment rate

Data on the duration (in days) from HIV diagnosis to ART initiation, $Z^{\{\ell\}}$, for years $\ell \in [2015..2019]$ is used to construct the time-dependent ART initiation rate $\zeta(t)$,

$$
\zeta(t) = \begin{cases} \frac{365}{Z^{\{2015\}}} & \text{for } t < 2015.5,\\[2ex] \frac{365}{Z^{\{\ell-1\}}} + (t-\ell+0.5)\left(\frac{365}{Z^{\{\ell\}}} - \frac{365}{Z^{\{\ell-1\}}}\right) & \text{for } \ell - 0.5 \le t < \ell + 0.5 \\ & \text{where } \ell \in [2016..2019],\\[2ex] \frac{365}{Z^{\{2019\}}} & \text{for } t \ge 2019.5. \end{cases} \tag{37}
$$

where we have assumed the 2015 treatment rate applies to the pre-outbreak era. Note that we have constructed $\zeta(t)$ so that the data on time from diagnosis to treatment initiation applies to the middle of the year in which it refers to.

2.6.4 Moving between testing and treatment compartments

For clarity, let $\gamma_{p,r,s}(t)$ denote the first systematic testing rate, which depends on homelessness status (p), OAT status (r) and geography status (s) and is defined as

$$
\begin{aligned}
\gamma_{p,r,1}(t) &= H^{test}_p(t) F^{test}_r(t)\alpha^{GCC}(t) + \beta^{GCC}(t),\\
\gamma_{p,r,2}(t) &= H^{test}_p(t) F^{test}_r(t)\alpha^{RoGGC}(t) + \beta^{RoGGC}(t).
\end{aligned} \tag{38}
$$

$K^{i,j,k}_{p,q,r,s}$ describes the movement between testing and treatment compartments. For 'susceptible' PWID:

$$\begin{aligned}
K^{i,1,1}_{p,q,r,s} &= -\gamma_{p,r,s}(t)Y^{i,1,1}_{p,q,r,s},\\
K^{i,1,2}_{p,q,r,s} &= \gamma_{p,r,s}(t)Y^{i,1,1}_{p,q,r,s},\\
K^{i,1,k}_{p,q,r,s} &= 0 \text{ for } k \in \{3,4\}.
\end{aligned} \tag{39}$$

For 'infected' PWID:

$$\begin{aligned}
K^{i,j,1}_{p,q,r,s} &= -\left(\gamma_{p,r,s}(t) + \epsilon(t)\chi\frac{D^{NCT}}{Y_I}\right)Y^{i,j,1}_{p,q,r,s},\\
K^{i,j,2}_{p,q,r,s} &= -\left(R^{repeat}\gamma_{p,r,s}(t) + \epsilon(t)\chi\frac{D^{NCT}}{Y_I}\right)Y^{i,j,2}_{p,q,r,s},\\
K^{i,j,3}_{p,q,r,s} &= \left(\gamma_{p,r,s}(t) + \epsilon(t)\chi\frac{D^{NCT}}{Y_I}\right)Y^{i,j,1}_{p,q,r,s} +\\
&\quad \left(R^{repeat}\gamma_{p,r,s}(t) + \epsilon(t)\chi\frac{D^{NCT}}{Y_I}\right)Y^{i,j,2}_{p,q,r,s} - \zeta(t)Y^{i,j,3}_{p,q,r,s},\\
K^{i,j,4}_{p,q,r,s} &= \zeta(t)Y^{i,j,3}_{p,q,r,s},
\end{aligned} \tag{40}$$

where $j \in [2..5]$.

We are assuming that people diagnosed with HIV don't undergo further testing.

2.7 Homelessness

The parameters for the homelessness component of the model are:

- ν^{init}_s: the rate at which PWID become homeless for the first time in GCC ($s = 1$) and RoGGC ($s = 2$),
- ν^{sub}_s: the rate at which PWID become homeless subsequent times in GCC ($s = 1$) and RoGGC ($s = 2$),
- ν^{out}: the rate at which PWID become housed.

We assume the rates to become homeless are geography dependent because the proportions ever homeless from NESI [5] differ between GCC and RoGGC.

The homelessness component of the model $P^{i,j,k}_{p,q,r,s}$ can be expressed as

$$\begin{aligned}
P^{i,j,k}_{1,q,r,s} &= -\nu^{init}_s Y^{i,j,k}_{1,q,r,s},\\
P^{i,j,k}_{2,q,r,s} &= \nu^{out}Y^{i,j,k}_{3,q,r,s} - \nu^{sub}_s Y^{i,j,k}_{2,q,r,s},\\
P^{i,j,k}_{3,q,r,s} &= \nu^{init}_s Y^{i,j,k}_{1,q,r,s} + \nu^{sub}_s Y^{i,j,k}_{2,q,r,s} - \nu^{out}Y^{i,j,k}_{3,q,r,s}.
\end{aligned} \tag{41}$$

2.8 Cocaine injecting

The rate for initiating cocaine injecting, $\xi_s(t)$, depends on geographical region (s). The rate in GCC is

$$\xi_1(t) = \begin{cases}
\xi^{pre}_1 & \text{if } t < 2015,\\
\xi^{pre}_1 + \left(\frac{t-2015}{2016-2015}\right)\left[\xi^{early}_1 - \xi^{pre}_1\right] & \text{if } 2015 \le t < 2016,\\
\xi^{early}_1 + \left(\frac{t-2016}{2018-2016}\right)\left[\xi^{mid}_1 - \xi^{early}_1\right] & \text{if } 2016 \le t < 2018,\\
\xi^{mid}_1 & \text{if } t \ge 2018.
\end{cases} \tag{42}$$

where ξ_1^{pre}, ξ_1^{early} and ξ_1^{mid} are the rates for initiating cocaine injecting in the pre-, early- and mid-outbreak periods respectively. The rate in RoGGC is

$$\xi_2(t) = \begin{cases} \xi_2^{pre} & \text{if } t < 2016, \\ \xi_2^{pre} + \left(\frac{t-2016}{2018-2016}\right)\left[\xi_2^{mid} - \xi_2^{pre}\right] & \text{if } 2016 \leq t < 2018, \\ \xi_2^{mid} + \left(\frac{t-2018}{2020-2018}\right)\left[\xi_2^{late} - \xi_2^{mid}\right] & \text{if } 2018 \leq t < 2020, \\ \xi_2^{late} & \text{if } t \geq 2020. \end{cases} \tag{43}$$

where ξ_2^{pre}, ξ_2^{mid} and ξ_2^{late} are the rates for initiating cocaine injecting in the pre-, mid- and late-outbreak periods respectively.

The difference in the structure of the time-dependence structure between the rates in GCC and RoGGC is due to the rise in cocaine injecting happening earlier in GCC compared to RoGGC.

The movement between cocaine injecting compartments is described by $Q_{p,q,r,s}^{i,j,k}$:

$$\begin{aligned} Q_{p,1,r,s}^{1,j,k} &= -\xi_s(t)Y_{p,1,r,s}^{1,j,k}, \\ Q_{p,2,r,s}^{1,j,k} &= \xi_s(t)Y_{p,1,r,s}^{1,j,k}, \\ Q_{p,1,r,s}^{2,j,k} &= -\xi_s(t)Y_{p,1,r,s}^{2,j,k}, \\ Q_{p,2,r,s}^{2,j,k} &= \xi_s(t)Y_{p,1,r,s}^{2,j,k}, \\ Q_{p,q,r,s}^{3,j,k} &= 0. \end{aligned} \tag{44}$$

where we have assumed that only PWID currently injecting initiate cocaine injecting.

2.9 OAT

The parameters relevant to OAT are:

- $\rho^{(1)}$: the rate for initiating OAT for the first time,
- $\rho^{(2)}$: the rate for initiating OAT for subsequent times,
- ρ^{off}: the rate for leaving OAT.

Then the OAT component of the model $R_{p,q,r,s}^{i,j,k}$ can be expressed as

$$\begin{aligned} R_{p,q,1,s}^{1,j,k} &= -\rho^{(1)}Y_{p,q,1,s}^{1,j,k}, \\ R_{p,q,2,s}^{1,j,k} &= \rho^{off}Y_{p,q,3,s}^{1,j,k} - \rho^{(2)}Y_{p,q,2,s}^{1,j,k}, \\ R_{p,q,3,s}^{1,j,k} &= \rho^{(1)}Y_{p,q,1,s}^{1,j,k} + \rho^{(2)}Y_{p,q,2,s}^{1,j,k} - \rho^{off}Y_{p,q,3,s}^{1,j,k}, \\ R_{p,q,1,s}^{2,j,k} &= -\rho^{(1)}Y_{p,q,1,s}^{2,j,k}, \\ R_{p,q,2,s}^{2,j,k} &= \rho^{off}Y_{p,q,3,s}^{2,j,k} - \rho^{(2)}Y_{p,q,2,s}^{2,j,k}, \\ R_{p,q,3,s}^{2,j,k} &= \rho^{(1)}Y_{p,q,1,s}^{2,j,k} + \rho^{(2)}Y_{p,q,2,s}^{2,j,k} - \rho^{off}Y_{p,q,3,s}^{2,j,k}, \\ R_{p,q,1,s}^{3,j,k} &= 0, \\ R_{p,q,2,s}^{3,j,k} &= \rho^{off}Y_{p,q,3,s}^{3,j,k}, \\ R_{p,q,3,s}^{3,j,k} &= -\rho^{off}Y_{p,q,3,s}^{3,j,k}, \end{aligned} \tag{45}$$

where we have assumed that PWID who have temporarily ceased injecting cannot initiate OAT.

2.10 Geography

We assume no movement between geography compartments because of the short time scale being modelled and lack of data to inform movement rates.

2.11 Submodel equations

Using data from the Edinburgh Addiction Cohort of opiate injectors, Xia et al. [24] calculated the following probabilities (conditional on survival for the specified time period):

- the probability of temporary cessation within 1 year of first injection,
- the probability of temporary cessation within 5 years of first injection,
- the probability of relapse within 5 years of first cessation,
- the probability of sustained cessation up to 30 years after first cessation.

We calibrate submodels to this data to estimate the temporary cessation, permanent cessation and relapse rates for PWID in the main model. The same rates are used for PWID in GCC and RoGGC.

2.11.1 Permanent cessation and relapse rates

Let y_i represent the proportion of PWID in each of the following injecting categories; 1=currently injecting, 2=temporarily ceased injecting, 3=permanently ceased injecting. The submodel equations are

$$\begin{aligned} \dot{y_1} &= \kappa^{inj} y_2, \\ \dot{y_2} &= -(\kappa^{inj} + \eta) y_2, \\ \dot{y_3} &= \eta y_2, \end{aligned} \tag{46}$$

where κ^{inj} and η are the relapse and permanent cessation rates respectively.

With initial conditions $(y_1, y_2, y_3) = (0, 1, 0)$, these equations have solution

$$\begin{aligned} y_1 &= \frac{\kappa^{inj}}{\kappa^{inj} + \eta}(1 - e^{-(\kappa^{inj}+\eta)t}), \\ y_2 &= e^{-(\kappa^{inj}+\eta)t}, \\ y_3 &= \frac{\eta}{\kappa^{inj} + \eta}(1 - e^{-(\kappa^{inj}+\eta)t}). \end{aligned} \tag{47}$$

The relevant probabilities can be calculated and compared with data;

$$\begin{aligned} \text{probability of relapse within 5 years} &= \frac{\kappa^{inj}}{\kappa^{inj} + \eta}(1 - e^{-5(\kappa^{inj}+\eta)}), \\ \text{probability of sustained cessation at 30 years} &= \frac{1}{\kappa^{inj} + \eta}(\eta + \kappa^{inj} e^{-30(\kappa^{inj}+\eta)}), \end{aligned} \tag{48}$$

where the probability of sustained cessation at 30 years is the sum of those who have permanently ceased at 30 years and those who remain temporarily ceased at 30 years.

Xia et al. [24] also calculated the probability of relapse within 1 year, but we don't calibrate to it. This is because the temporary ceased category would need to be further stratified by duration temporarily ceased to accurately calibrate to all of the data. The paper by Xia et al. [24] suggests that this stratification is less important than stratifying the currently injecting population based on duration currently injecting. Furthermore, sensitivity analysis suggests that the relapse rate has a small impact on the modelling results. Given the time scale of the model, we decided it was more important to calibrate to the 5 year relapse value compared to the 1 year value.

2.11.2 Temporary cessation rates

Let $y_{i,j}$ represent the proportion of PWID in each category where; $i \in \{1, 2\}$ (1=currently injecting, 2=temporarily ceased injecting) and $j \in \{1, 2\}$ (1 = not on OAT, 2 = on OAT).

The parameters in the submodel are:

- ρ_{SM}^{on}: the rate for initiating OAT in the submodel,
- ρ_{SM}^{off}: the rate for leaving OAT in the submodel,
- $\kappa^{(c1)}$: the rate at which currently injecting (< 1 yr) PWID temporarily cease injecting,
- $\kappa^{(c2)}$: the rate at which currently injecting ($\geq$ 1 yr) PWID temporarily cease injecting,
- F_3^{cease}: the effect of being on OAT on the rate of temporary cessation of injecting, defined in Section 2.4.

The OAT leaving rate is the same as in the main model; $\rho_{SM}^{off} = \rho^{off}$. The rate for initiating OAT is calculated so that the overall proportion of PWID on OAT in the submodel remains constant; $\rho_{SM}^{on} = \frac{p^{OAT}}{1-p^{OAT}} \rho_{SM}^{off}$, where p^{OAT} is the proportion of PWID on OAT in the submodel.

The equations for this submodel are:

$$\begin{aligned} \dot{y}_{1,1} &= \begin{cases} \rho_{SM}^{off} y_{1,1} - \rho_{SM}^{on} y_{1,2} - \kappa^{(c1)} y_{1,1} & \text{if } t < 1, \\ \rho_{SM}^{off} y_{1,1} - \rho_{SM}^{on} y_{1,2} - \kappa^{(c2)} y_{1,1} & \text{if } t \geq 1, \end{cases} \\ \dot{y}_{1,2} &= \begin{cases} -\rho_{SM}^{off} y_{1,1} + \rho_{SM}^{on} y_{1,2} - F_3^{cease} \kappa^{(c1)} y_{1,2} & \text{if } t < 1, \\ -\rho_{SM}^{off} y_{1,1} + \rho_{SM}^{on} y_{1,2} - F_3^{cease} \kappa^{(c2)} y_{1,2} & \text{if } t \geq 1. \end{cases} \end{aligned} \tag{49}$$

After solving these equations, the relevant probabilities can be calculated and compared with the data,

$$\begin{aligned} \text{probability of cessation within 1 year} &= 1 - (y_{1,1}(1) + y_{1,2}(1)), \\ \text{probability of cessation within 5 years} &= 1 - (y_{1,1}(5) + y_{1,2}(5)). \end{aligned} \tag{50}$$

2.12 Additional model details

2.12.1 Model complexity

To minimise model complexity, certain aspects of the outbreak and response were not included. We have not explicitly modelled sexual transmission of HIV because high prevalence of hepatitis C virus among the outbreak cohort suggests transmission via sharing of injecting equipment [20]. Incarceration dynamics were not included because the data from the Needle Exchange Surveillance Initiative (NESI) surveys suggests that cocaine and homelessness are more important risk factors to include [15, 5]. The impact of needle and syringe programmes (NSP) was not explicitly included in the model because provision remained steady over the course of the outbreak [15, 5].

2.12.2 Inconsistency in the data

There are inconsistencies in the data related to HIV prevalence [5], HIV diagnoses [1] and estimates for the PWID population size size in Glasgow [9]. In the model, the population size is calibrated from a wide prior distribution to the available data on HIV prevalence and diagnoses, which results in a smaller population size than the estimates reported in the literature. As discussed in the main text, this is likely due to differences in the modelled population and the target population for the PWID population size estimates; we are not modelling the lowest risk category of people who have ever injected drugs. For example, the modelled population doesn't include those who have 'permanently ceased' injecting which may include people who have ever injected drugs who are on OAT, but in a stable state of abstinence from injecting. However, these people may still be engaged with services and therefore being tested for HIV. If no adjustment is made, the smaller population size assumed in the model may result in a higher proportion of HIV tests per person than reality. To account for this, we adjusted the function used to compare model outputs with data when calibrating the model; model predictions within $50\% - 100\%$ of the data value are considered an equally

good fit. This leads to wider posterior distributions for the testing rates. This uncertainty propagates to the model results and is reflected in the reported credibility intervals. Due to the uncertainties in the PWID population size, the model outputs for the relative changes in prevalence/incidence/new cases are likely to be more accurate than absolute values. However, we still have confidence in these results because we have captured those who are highest risk.

2.12.3 HIV testing

HIV testing is a multi-faceted intervention. The data used to parameterise and calibrate this part of the model came from multiple sources, as detailed in Tables S1 and S2. However, there are gaps which have necessarily required assumptions to be made when carrying out the modelling.

We have annual data for the number of HIV tests carried out in drugs services in GGC. We assume that all of these tests were carried out among PWID (either currently or temporarily ceased injecting). However, an appreciable proportion of HIV diagnoses were made in other locations [1]. This was accounted for in the model by using the data on the location of last HIV test from NESI [5]. However, this data was only available for the 2019-2020 survey, so we had to make assumptions about how the testing rates in locations other than drugs services changed over time. In the model, we included a factor describing the time dependence of tests in other locations relative to tests in drugs services, which was included in the model and calibrated to estimates for the proportion of tests in other locations based on HIV diagnoses. These estimates were constructed from data on the proportion of diagnoses which occurred in drugs services or prisons each year, the total number of tests carried out in drugs services each year and the total number of tests carried out in prisons each year. These estimates have wide confidence intervals due to the small number of HIV diagnoses. Sensitivity analysis was carried out on the time-dependence factor to see how the overall results are affected.

In the model we have assumed different rates for first and repeat HIV tests. We assumed a constant factor to account for the difference in first/repeat testing rates due to lack of data on how this may have changed over time.

We have assumed the same testing rates for PWID currently injecting and PWID who have temporarily ceased injecting. It is assumed that PWID who are temporarily ceased still engage with services (e.g. when collecting OAT prescriptions), so will still have access to HIV testing.

There is limited data on how contact tracing was implemented over time. We assumed contact tracing started 3 months into 2015 and was fully operational 6 months into 2015, and assumed a wide prior distribution for the contact tracing rate. This led to the model predicting 32.2% (95%CrI 21.8-42.2) of PWID diagnosed with HIV were diagnosed through contact tracing by the start of 2020, which is consistent with estimates given by those involved in contact tracing [17].

2.12.4 Treatment

Unlike with testing, the treatment data all came from one source as it was implemented as part of one initiative, Glasgow Enhanced Care HIV Outreach (GECHO) [17]. This data gave a detailed account of how treatment rates and viral suppression changed over time for the whole outbreak cohort, consisting of all those diagnosed with HIV as part of the outbreak. However, due to the relatively small number of people in the outbreak cohort, there is insufficient data to determine whether certain groups of people have higher or lower treatment rates. Therefore, we assume that the treatment rate is the same for all individuals once diagnosed with HIV. Note that differences in testing rates, which have been incorporated into the model, will impact time from seroconversion to ART initiation.

2.12.5 OAT

There isn't sufficient information about how OAT affects people who inject cocaine. We assume that OAT reduces HIV transmission/acquisition of PWID who inject cocaine because most PWID who injecting cocaine in Glasgow also inject opiates [5]. We have also assumed that OAT does not affect the cessation rates for PWID who inject cocaine because we assume OAT has no impact on the frequency of cocaine injecting.

3 Parameterisation and calibration

3.1 The calibration algorithm

An Approximate Bayesian Computation Sequential Monte Carlo (ABC SMC) scheme was used to calibrate the model. We utilised the algorithm presented by Toni et al. [22]. A number of parameter sets are initially constructed by sampling each parameter from prior distributions. The parameters are propagated through a series of intermediate distributions before a target posterior distribution is reached. At each iteration, each parameter set is given a score based on how closely it fits the data, measured using a distance function. The scores are ranked and a tolerance is calculated such that a certain proportion of the model fits have a score within the tolerance. At the next iteration, parameter sets are constructed by sampling from the previous parameter sets, perturbing by a certain proportion, and checking the score lies within the required tolerance. This process is repeated until a stopping criteria is met. The distributions of the parameters in the final parameter sets are referred to as the posterior distributions. The ABC SMC hyperparameter values used are:

- Number of parameter sets: 7500
- Perturbation: 0.1
- Proportion of model runs used to define the tolerance for the next iteration: 0.75
- Distance function:
  - For the main model: $\sum_{i \in \mathcal{D}_{HIV}} 2|\frac{m_i - d_i}{n_i}| + \sum_{i \in \mathcal{D}_{other}} |\frac{m_i - d_i}{n_i}|$ where d_i are the data points, m_i are the model outputs and n_i is a normalising factor. If d_i is not a proportion, $n_i = d_i$. Otherwise, $n_i = \min(d_i, 1 - d_i)$. $\mathcal{D}_{HIV}$ refers to the indices of data points related to HIV and $\mathcal{D}_{other}$ refers to the indices of all other data points. For the number of HIV tests, the score is zero unless outside of the data's error bounds, in which case it the score is as specified here.
  - For the submodels: If model estimate is within the data's error bounds: 0; Otherwise: $\sum_{i \in \mathcal{D}_{sub}} |\frac{m_i - d_i}{n_i}|$ where d_i are the data points, m_i are the model outputs and n_i is a normalising factor as defined above. $\mathcal{D}_{sub}$ refers to the indices of data points related to the submodels.
- Stopping criteria: The distance between model outputs and the calibration data is ≤ 36.6, measured using the distance function defined above.

Note that the distance function for the main model is weighted in favour of HIV prevalence data. This is required to capture the shape of the outbreak. The HIV weighting was chosen so that the profile of HIV prevalence and diagnoses was correct over time, without overly compromising on fit to other data.

A stopping criteria which uses a fixed final tolerance has been used elsewhere [6]. The final tolerance is equal to half of the value of the score corresponding to the upper/lower limits of the calibration data. For the ABC SMC hyperparameters used, successive tolerances are within approximately 99% of each other when the algorithm stops.

3.2 Further calibration details

As well as calibrating to the data in Table S2, we also calibrate to additional quantities based on assumptions we're making about rates in the model. We calculate the rate of change over time of the proportions injecting cocaine in GCC and RoGGC and the proportions who have ever had an HIV test in GCC, RoGGC, and among people in each homelessness and OAT category. We calibrate these rates of change over time to a value of zero in 2013.5 (the time point used to represent the pre-outbreak era). This is because we assume that the proportions of PWID who inject cocaine and the proportions who have ever had an HIV test are in a steady state before the outbreak. Additionally, where we're calibrating to data in the pre-, early-, mid- and late-outbreak eras, we also calibrate to the value of the late-outbreak era data point in 2022 to encourage the system to tend towards a steady state in the late-outbreak era.

For calibration purposes, the model was run from 2011 to 2022. This allows a two-year burn-in period before the start of the pre-outbreak era (2013-2014), and allows us to encourage the system to reach a steady state in the late-outbreak era (2019-2020) by calibrating to late-outbreak data in 2022.

3.2.1 Cocaine injecting prior distributions

Initially, we used wide uninformed prior distributions for the initiating cocaine injecting rates. However, calibrating with wide prior distributions led to wide posterior distributions because the proportion of PWID injecting cocaine could hold values in a wide range whilst still fitting well to the rest of the calibration data. To encourage a better fit to the cocaine injecting data, the prior distributions were informed by calibrating a version of the model to the cocaine injecting data only. Sensitivity analysis shows that the model results are not sensitive to changes in these parameters.

3.3 Parameter and calibration data tables

Table S1: Parameter table detailing parameter values and distributions used to calibrate the model. Outbreak eras are defined as: Pre = 2013-2014, Early = 2015-2016, Mid = 2017-2018, Late = 2019-2020. Q1-Q3 refers to quartiles 1 to 3.

Description	Symbol*	Value	Distribution	Source and notes	Posteriors - median (range)
**Population size**					
Population size	N	Range 2600 - 11400	Uniform	The lower bound is calculated using data related to HIV prevalence [5], the number of HIV diagnoses [17] and an estimate for the proportion of people currently injecting [5]. The upper bound is taken from an estimate for the PWID population size in GGC calculated by Hay et al. [9].	3230 (2600 - 5130)
**Entering and leaving the model**					
Drug related mortality rate (per 1000 PY).	$\omega^{DRD}(t)$	2009-2013: Range 4.8 - 7.2<br>2018: Range 11.1 - 13.8	Uniform	[16]	2013: 6.46 (4.81 - 7.20)<br>2018: 12.5 (11.1 - 13.8)
Non drug related mortality rate (per 1000 PY).	ω^{nDRD}	10.0 (95% CI 9.5 - 10.5)	Truncated normal	[8]	10.0 (9.59 - 10.5)
The proportion entering in each homelessness category.	$p^H_{p,s}$. Note that $p^H_{2,s}$ is $1 - p^H_{1,s} - p^H_{3,s}$.	Proportion entering as never homeless:<br>In GCC: Range 0.01 - 0.49; In RoGGC: Range 0.30 - 0.61.<br>Proportion entering as homeless in the last 6 months:<br>In GCC: Range 0.22 - 0.78; In RoGGC: 0.20 - 0.49.	Uniform	NESI [5]	Proportion entering as never homeless:<br>In GCC: 0.27 (0.03 - 0.49)<br>In RoGGC: 0.52 (0.30 - 0.61)<br>Proportion entering as homeless in the last 6 months:<br>In GCC: 0.49 (0.22 - 0.78)<br>In RoGGC: 0.37 (0.21 - 0.49)

Table S1: Parameter table detailing parameter values and distributions used to calibrate the model. Outbreak eras are defined as: Pre = 2013-2014, Early = 2015-2016, Mid = 2017-2018, Late = 2019-2020. Q1-Q3 refers to quartiles 1 to 3. *(continued)*

Description	Symbol*	Value	Distribution	Source and notes	Posteriors - median (range)
Proportion of PWID entering in each cocaine injecting category.	$p^C_{q,s}$. Note that $p^C_{1,s} = 1 - p^C_{2,s}$.	Proportion entering as cocaine injectors:<br>-pre/early:<br>In GCC: 0 (fixed value); In RoGGC: Range 0.00 - 0.21<br>-mid/late:<br>In GGC: Range 0.36 - 1.00; In RoGGC Range 0.32 - 0.77	Uniform	NESI [5]	Proportion entering as cocaine injectors:<br>-pre/early:<br>In RoGGC: 0.11 (0.00 - 0.21)<br>-mid/late:<br>In GGC: 0.71 (0.36 - 0.99)<br>In RoGGC: 0.53 (0.32 - 0.77)
Proportion of PWID entering in each geography category.	p^G_s. Note that $p^G_2 = 1 - p^G_1$.	Proportion entering into RoGGC: Range 0.66 - 0.83 (Same as the initial proportion in RoGGC)	Uniform	NESI [5]	0.78 (0.66 - 0.83)
**Injecting submodels**					
Rate for permanent cessation of injecting.	η	0.00 - 0.15	Uniform	Uninformed prior	0.09 (0.05 - 0.12)
Rate at which PWID move from temporarily ceased injecting to current injecting.	κ^{inj}	0.15 - 0.35	Uniform	Uninformed prior	0.25 (0.19 - 0.30)
Rate at which PWID move from current injecting (< 1 yr) to temporarily ceased injecting.	$\kappa^{(c1)}$	0.3 - 0.6	Uniform	Uninformed prior	0.46 (0.35 - 0.57)
Rate at which PWID move from current injecting (>1 yr) to temporarily ceased injecting.	$\kappa^{(c2)}$	0.05 - 0.2	Uniform	Uninformed prior	0.13 (0.09 - 0.17)
Proportion on OAT in injecting submodels.	p^{OAT}	0.7	Fixed value	Based on proportion recently on OAT in Edinburgh Addiction Cohort [12], which was the data used by Xia et al. [24] to estimate probabilities of cessation and relapse.	N/A

Table S1: Parameter table detailing parameter values and distributions used to calibrate the model. Outbreak eras are defined as: Pre = 2013-2014, Early = 2015-2016, Mid = 2017-2018, Late = 2019-2020. Q1-Q3 refers to quartiles 1 to 3. *(continued)*

Description	Symbol*	Value	Distribution	Source and notes	Posteriors - median (range)
**Disease progression and transmission**					
Duration of the acute stage of infection (months).	Use to calculate $\tau^{(1)}$: Rate of progression from the acute stage of infection.	2.90 (95% CI 1.23 - 6.00)	Truncated lognormal	[10]	2.20 (1.23 - 3.83)
Time from infection to AIDS (years).	Used to calculate $\tau^{(2)}$: Rate of progression from the latent stage of infection.	Range 7.1 - 11.7.	Uniform	[2]	9.87 (7.10 - 11.7)
Duration of pre-AIDS phase of infection (months)	Used to calculate $\tau^{(3)}$: Rate of progression from the pre-AIDS stage of infection.	9.00 (95% CI 4.81 - 14.0)	Truncated lognormal	[10]	8.39 (4.83 - 12.9)
Duration of AIDS phase of infection (months).	Used to calculate $\tau^{(4)}$: AIDS mortality rate.	10.0 (95% CI 6.97 - 12.7)	Truncated lognormal	[10]	9.72 (7.01 - 12.5)
Per capita HIV transmission rate.	λ	Range 0.00 - 0.30	Uniform	Uninformed prior	0.11 (0.05 - 0.22)
HIV transmissability from PWID with acute infection.	$\psi^{(1)}$	276 (95% CI 131 - 509)	Truncated lognormal	[10]	204 (131 - 334)
HIV transmissability from PWID with latent infection.	$\psi^{(2)}$	10.6 (95% CI 7.61 - 13.3)	Truncated lognormal	[10]	11.0 (8.47 - 13.3)
HIV transmissability from PWID with pre-AIDS infection.	$\psi^{(3)}$	76.0 (95% CI 41.3 - 128)	Truncated lognormal	[10]	67.5 (41.5 - 105)
Proportion of assortative mixing according to homelessness status.	σ^H	Range 0.00 - 1.00	Uniform	Uninformed prior	0.46 (0.00 - 0.96)
Proportion of assortative mixing according to cocaine injecting status.	σ^C	Range 0.00 - 1.00	Uniform	Uninformed prior	0.75 (0.25 - 0.99)
Proportion of assortative mixing according to geography status.	σ^G	Range 0.00 - 1.00	Uniform	Uninformed prior	0.76 (0.26 - 1.00)

Table S1: Parameter table detailing parameter values and distributions used to calibrate the model. Outbreak eras are defined as: Pre = 2013-2014, Early = 2015-2016, Mid = 2017-2018, Late = 2019-2020. Q1-Q3 refers to quartiles 1 to 3. *(continued)*

Description	Symbol*	Value	Distribution	Source and notes	Posteriors - median (range)
**Testing and treatment**					
Contact tracing rate.	ϵ	12 (Range 0 - 52)	Triangular	Uninformed prior; peak value chosen to correspond to average time from diagnosis to contact of 1 month.	15.9 (1.01 - 35.6)
Average number of HIV+ contacts from one person, adjusted for contact tracing success.	χ	Range 1.00 - 3.00	Uniform	Guided by contact tracing data [17]	2.03 (1.00 - 3.00)
Ratio of repeat to first testing rate.	R^{repeat}	Range 1.00 - 4.00	Uniform	Uninformed prior	1.72 (1.01 - 3.14)
Proportion of HIV tests in other locations in 2019 which follow the same time dependence as HIV tests in drug services.	q	Range 0.00 - 1.00	Uniform	Uninformed prior	0.72 (0.01 - 1.00)
Proportion of current injectors who had their last HIV test at drug services (DS).	w_s^{OL}	In GCC: Range 0.38 - 0.56<br>In RoGGC: Range 0.53 - 0.62	Uniform	NESI [5]	In GCC: 0.51 (0.39 - 0.56)<br>In RoGGC: 0.58 (0.53 - 0.62)
Proportion of HIV+ PWID on ART who are virally suppressed.	$\phi(t)$	Pre mid-2016: Range 0.61 - 0.68<br>Post mid-2018: Range 0.84 - 0.89	Uniform	[17, 3]	Pre: 0.65 (0.61 - 0.68)<br>Post: 0.87 (0.84 - 0.89)
Increase in HIV transmissibility per $\log_{10}$ increase in HIV viral load	υ	2.45 (95% CI 1.85 - 3.26)	Truncated lognormal	[19]	2.42 (1.88 - 3.02)
Log (base 10) difference between the PVL at the start of ART (copies/ml) and the PVL after 1.5 yrs for those virally suppressed (copies/ml).	Δ^S	Median 5.311 (Q1-Q3 3.899 - 6.620)	Triangular	[14]	5.26 (3.91 - 6.37)

Table S1: Parameter table detailing parameter values and distributions used to calibrate the model. Outbreak eras are defined as: Pre = 2013-2014, Early = 2015-2016, Mid = 2017-2018, Late = 2019-2020. Q1-Q3 refers to quartiles 1 to 3. *(continued)*

Description	Symbol*	Value	Distribution	Source and notes	Posteriors - median (range)
Log (base 10) difference between the PVL at the start of ART (copies/ml) and the PVL after 1.5 yrs for those not virally suppressed (copies/ml).	Δ^U	Median 1.577 (Q1-Q3 -0.231 - 5.030)	Triangular	[14]	1.94 (0.11 - 4.42)
Time from HIV diagnosis to ART initiation (days) by year.	$Z^{\{\ell\}}$, where $\ell \in [2015..2019]$ represents the year. Used to calculate ζ: A time dependent rate for initiating ART treatment.	2015: 264 (Q1-Q3 94 - 556)<br>2016: 139 (Q1-Q3 49 - 280)<br>2017: 73 (Q1-Q3 25 - 156)<br>2018: 28 (Q1-Q3 18 - 51)<br>2019: 23 (Q1-Q3 12 - 38)	Triangular	[17, 3]	2015: 298 (114 - 500)<br>2016: 143 (55.7 - 238)<br>2017: 75.2 (25.7 - 147)<br>2018: 28.0 (18.5 - 43.4)<br>2019: 23.3 (12.7 - 36.3)
Factor for the effect of ART on HIV disease progression.	T_k^{prog}. Note that $T_1^{prog} = T_2^{prog} = T_3^{prog} = 1$.	Range 0.08 - 0.46	Uniform	[18]	0.26 (0.08 - 0.46)
Rate at which PWID who are not homeless, not on OAT and who live in GCC get first HIV test from drug services.	$\alpha^{GCC}(t)$	Rate in pre-outbreak era: Range 0.00 - 0.25<br>Increase in early-outbreak era: Range 0.00 - 0.50<br>Increase in mid-outbreak era: Range 0.00 - 0.50<br>Increase in late-outbreak era: Range 0.00 - 0.50	Uniform	Uninformed prior	Rate in pre-outbreak era: 0.09 (0.00 - 0.21)<br>Increase in early outbreak era: 0.20 (0.00 - 0.49)<br>Increase in mid outbreak era: 0.13 (0.00 - 0.49)<br>Increase in late outbreak era: 0.19 (0.00 - 0.49)
Rate at which PWID who are not homeless, not on OAT and who live in RoGGC get first HIV test from drug services.	$\alpha^{RoGGC}(t)$	Rate in pre-outbreak era: Range 0.00 - 0.25<br>Increase in early-outbreak era: Range 0.00 - 0.25<br>Increase in mid-outbreak era: Range 0.00 - 0.25<br>Increase in late-outbreak era: Rnage 0.00 - 0.25	Uniform	Uninformed prior	Rate in pre-outbreak era: 0.11 (0.04 - 0.21)<br>Increase in early outbreak era: 0.07 (0.00 - 0.20)<br>Increase in mid outbreak era: 0.06 (0.00 - 0.24)<br>Increase in late outbreak era: 0.06 (0.00 - 0.21)

Table S1: Parameter table detailing parameter values and distributions used to calibrate the model. Outbreak eras are defined as: Pre = 2013-2014, Early = 2015-2016, Mid = 2017-2018, Late = 2019-2020. Q1-Q3 refers to quartiles 1 to 3. *(continued)*

Description	Symbol*	Value	Distribution	Source and notes	Posteriors - median (range)
**Risk**					
Factor for the relative effect of homelessness on transmission.	H_p^{inf}. Note that $H_1^{inf} = H_2^{inf} = 1$.	Range 1.00 - 3.00	Uniform	Uninformed prior	2.21 (1.26 - 3.00)
Factor for the relative effect of cocaine injecting on transmission.	C_q^{inf}. Note that $C_1^{inf} = 1$.	Range 1.00 - 5.00	Uniform	Uninformed prior	3.12 (1.30 - 4.99)
Factor describing the effect of homelessness on testing rates. Different effects are assumed pre/post targeted testing intervention.	$H_p^{test}(t)$ Note that $H_1^{test} = H_2^{test} = 1$.	Pre mid-2016: 1.00 (fixed value)<br>Post mid-2017: Range 1.00 - 2.50	Uniform	Uninformed prior	Post: 1.27 (1.00 - 2.08)
The proportion of homeless PWID who are housed after 8 months. Used to calculate the rate out of homelessness, ν^{out}.	$1 - e^{-\frac{2}{3}\nu^{out}}$	Range (given as fractions so it's more clear where data came from)<br>$\frac{118}{313} = 0.38 - \frac{118}{211} = 0.56$	Uniform	[11]	0.42 (0.38 - 0.52)
Rate for becoming homeless the first time for those in GCC.	ν_s^{init}	In GGC: Range 0.00 - 0.80<br>In RoGGC: Range 0.00 - 0.30	Uniform	Uninformed prior	In GGC: 0.13 (0.00 - 0.35)<br>In RoGGC: 0.07 (0.00 - 0.21)
Rate for becoming homeless subsequent times for those in GCC.	ν_s^{sub}	In GGC: Range 0.00 - 5.00<br>In RoGGC: Range 0.00 - 0.60	Uniform	Uninformed prior	In GGC: 2.16 (0.67 - 4.99)<br>In RoGGC: 0.22 (0.04 - 0.43)
Rate at which individuals start injecting cocaine.	ξ_s is a piecewise smooth function of time constructed from data for the rate at the mid-points of each outbreak period.	In GCC:<br>- pre: Range 0.00 - 0.20<br>- early: Range 0.00 - 2.00<br>- mid: Range 0.00 - 0.50<br>In RoGGC:<br>- pre: Range 0.00 - 0.06<br>- mid: Range 0.00 - 0.60<br>- late: Range 0.00 - 0.15	Uniform	Prior distribution informed by calibrating a version of the model to cocaine injecting data only.	In GCC:<br>- pre: 0.11 (0.00 - 0.20)<br>- early: 0.65 (0.01 - 1.63)<br>- mid: 0.23 (0.04 - 0.50)<br>In RoGGC:<br>- pre: 0.02 (0.00 - 0.04)<br>- mid: 0.27 (0.00 - 0.59)<br>- late: 0.07 (0.01 - 0.15)

Table S1: Parameter table detailing parameter values and distributions used to calibrate the model. Outbreak eras are defined as: Pre = 2013-2014, Early = 2015-2016, Mid = 2017-2018, Late = 2019-2020. Q1-Q3 refers to quartiles 1 to 3. *(continued)*

Description	Symbol*	Value	Distribution	Source and notes	Posteriors - median (range)
**OAT**					
Factor describing the effect of OAT on the rate of temporarily ceasing injecting.	F_r^{cease}. Note that $F_1^{cease} = F_2^{cease} = 1$.	Range 1.40 - 2.09	Uniform	[24]	1.54 (1.40 - 1.85)
Factor describing the effect of OAT on HIV disease transmission.	F_r^{inf}. Note that $F_1^{inf} = F_2^{inf} = 1$.	Mean 0.46 (95% CI 0.32 - 0.67)	Truncated normal	[13]	0.47 (0.33 - 0.63)
Rate of recruitment for those starting OAT for the first time (per PY).	$\rho^{(1)}$	Range 0.00 - 2.00	Uniform	Uninformed prior	0.93 (0.53 - 1.46)
Rate of recruitment for those starting OAT for subsequent times (per PY).	$\rho^{(2)}$	Range 2.00 - 12.00	Uniform	Uninformed prior; upper bound assumes prescriptions are once a month, so can't be 'off OAT' for less than a month.	8.37 (1.47 - 12.0)
OAT leaving rate (per PY).	ρ^{off}	0.00 - 2.00	Uniform	Uninformed prior; upper bound fixed due to definition of 'recently on OAT', people are considered to be on OAT for at least 6 months.	0.61 (0.00 - 1.82)
Factor describing the effect of OAT on testing rates.	$F_r^{test}(t)$. Note that $F_1^{test} = F_2^{test} = 1$.	Pre mid-2015: Range 1.00 - 2.50<br>Post mid-2016: Range 1.00 - 3.00	Uniform	Uninformed prior	Pre: 1.27 (1.00 - 1.98)<br>Post: 1.65 (1.04 - 2.50)

* *Indices:*
i: 1,2 = currently injecting, 3=temporarily ceased;
j : 1 = Susceptible to HIV, 2 = HIV - acute, 3 = HIV - latent, 4 = HIV - pre-AIDS, 5 = HIV - AIDS;
k : 1 =undiagnosed, never tested, 2 =undiagnosed, ever tested, 3 =diagnosed, not on ART, 4 =diagnosed, on ART;
p: 1=never homeless, 2=previously but not recently homeless, 3=recently homeless;
q: 1=never injected cocaine, 2=injects cocaine;
r: 1=Never on OAT, 2=Previously on OAT, 3=On OAT;
s: 1=GCC, 2=RoGGC

Table S2: Data used to calibrate the model. Outbreak eras are defined as: Pre = 2013-2014, Early = 2015-2016, Mid = 2017-2018, Late = 2019-2020.

Description	Value	Source	Posteriors
**Injecting submodels**			
The probability of temporary cessation of injecting at 12 months among current injectors).	0.46 (95% CI 0.41 - 0.50)	[24]	0.45 (95% CrI 0.41 - 0.49)
The probability of temporary cessation of injecting at 5 years among current injectors.	0.72 (95% CI 0.67 - 0.76)	[24]	0.74 (95% CrI 0.69 - 0.76)
The probability of replasing after 5 years.	0.59 (95% CI 0.54 - 0.64)	[24]	0.59 (95% CrI 0.55 - 0.63)
The probability of sustained cessation after 25 years.	≈ 0.25 with error bounds 0.20 - 0.30	Estimated from fig. 3 in [24]	0.27 (95% CrI 0.22 - 0.30)
**Disease progression and transmission**			
Proportion with HIV among PWID who have never been homeless	Pre: 0.024 (95% CI 0.001 - 0.047)<br>Early: 0.013 (95% CI 0.000 - 0.029)<br>Mid: 0.031 (95% CI 0.001 - 0.060)<br>Late: 0.015 (95% CI 0.000 - 0.033)	NESI [5]	Pre: 0.00 (95% CrI 0.00 - 0.00)<br>Early: 0.01 (95% CrI 0.01 - 0.01)<br>Mid: 0.02 (95% CrI 0.01 - 0.03)<br>Late: 0.02 (95% CrI 0.01 - 0.03)
Proportion with HIV among PWID who have previously been homeless but are not recently homeless	Pre: 0.003 (95% CI 0.000 - 0.009)<br>Early: 0.011 (95% CI 0.000 - 0.022)<br>Mid: 0.047 (95% CI 0.021 - 0.073)<br>Late: 0.038 (95% CI 0.016 - 0.061)	NESI [5]	Pre: 0.00 (95% CrI 0.00 - 0.01)<br>Early: 0.02 (95% CrI 0.01 - 0.02)<br>Mid: 0.04 (95% CrI 0.03 - 0.05)<br>Late: 0.05 (95% CrI 0.04 - 0.07)
Proportion with HIV among PWID who are recently homeless	Pre: 0.010 (95% CI 0.000 - 0.023)<br>Early: 0.090 (95% CI 0.044 - 0.135)<br>Mid: 0.113 (95% CI 0.065 - 0.161)<br>Late: 0.123 (95% CI 0.080 - 0.166)	NESI [5]	Pre: 0.01 (95% CrI 0.01 - 0.01)<br>Early: 0.04 (95% CrI 0.03 - 0.06)<br>Mid: 0.09 (95% CrI 0.07 - 0.12)<br>Late: 0.10 (95% CrI 0.08 - 0.13)
Proportion with HIV among PWID who have not injected cocaine in the last 6 months	Pre: 0.006 (95% CI 0.000 - 0.012)<br>Early: 0.004 (95% CI 0.000 - 0.009)<br>Mid: 0.022 (95% CI 0.004 - 0.039)<br>Late: 0.022 (95% CI 0.006 - 0.039)	NESI [5]	Pre: 0.00 (95% CrI 0.00 - 0.00)<br>Early: 0.00 (95% CrI 0.00 - 0.01)<br>Mid: 0.01 (95% CrI 0.00 - 0.02)<br>Late: 0.01 (95% CrI 0.00 - 0.02)
Proportion with HIV among PWID who have injected cocaine in the last 6 months	Pre: 0.024 (95% CI 0.001 - 0.047)<br>Early: 0.102 (95% CI 0.058 - 0.145)<br>Mid: 0.104 (95% CI 0.068 - 0.140)<br>Late: 0.089 (95% CI 0.060 - 0.116)	NESI [5]	Pre: 0.02 (95% CrI 0.01 - 0.03)<br>Early: 0.08 (95% CrI 0.05 - 0.10)<br>Mid: 0.11 (95% CrI 0.09 - 0.14)<br>Late: 0.10 (95% CrI 0.08 - 0.12)

Table S2: Data used to calibrate the model. Outbreak eras are defined as: Pre = 2013-2014, Early = 2015-2016, Mid = 2017-2018, Late = 2019-2020. *(continued)*

Description	Value	Source	Posteriors
Proportion with HIV among those in GCC	Pre: 0.009 (95% CI 0.000 - 0.022)<br>Early: 0.075 (95% CI 0.035 - 0.114)<br>Mid: 0.149 (95% CI 0.091 - 0.207)<br>Late: 0.140 (95% CI 0.082 - 0.197)	NESI [5]	Pre: 0.01 (95% CrI 0.01 - 0.02)<br>Early: 0.06 (95% CrI 0.04 - 0.08)<br>Mid: 0.13 (95% CrI 0.10 - 0.17)<br>Late: 0.15 (95% CrI 0.11 - 0.18)
Proportion with HIV among those in RoGGC	Pre: 0.010 (95% CI 0.001 - 0.019)<br>Early: 0.014 (95% CI 0.004 - 0.024)<br>Mid: 0.032 (95% CI 0.015 - 0.049)<br>Late: 0.039 (95% CI 0.023 - 0.055)	NESI [5]	Pre: 0.00 (95% CrI 0.00 - 0.01)<br>Early: 0.01 (95% CrI 0.01 - 0.02)<br>Mid: 0.03 (95% CrI 0.02 - 0.03)<br>Late: 0.03 (95% CrI 0.02 - 0.04)
**Testing and treatment**			
Proportion who have ever had an HIV test among PWID who have never been homeless	Pre: 0.69 (95% CI 0.62 - 0.76)<br>Early: 0.81 (95% CI 0.76 - 0.87)<br>Mid: 0.78 (95% CI 0.71 - 0.85)<br>Late: 0.84 (95% CI 0.79 - 0.89)	NESI [5]	Pre: 0.74 (95% CrI 0.70 - 0.79)<br>Early: 0.75 (95% CrI 0.69 - 0.80)<br>Mid: 0.80 (95% CrI 0.76 - 0.84)<br>Late: 0.84 (95% CrI 0.79 - 0.88)
Proportion who have ever had an HIV test among PWID who have previously been homeless but are not recently homeless	Pre: 0.83 (95% CI 0.79 - 0.87)<br>Early: 0.89 (95% CI 0.86 - 0.93)<br>Mid: 0.93 (95% CI 0.90 - 0.96)<br>Late: 0.95 (95% CI 0.92 - 0.97)	NESI [5]	Pre: 0.84 (95% CrI 0.81 - 0.87)<br>Early: 0.87 (95% CrI 0.84 - 0.90)<br>Mid: 0.92 (95% CrI 0.90 - 0.94)<br>Late: 0.95 (95% CrI 0.93 - 0.96)
Proportion who have ever had an HIV test among PWID who are recently homeless	Pre: 0.81 (95% CI 0.76 - 0.87)<br>Early: 0.86 (95% CI 0.80 - 0.91)<br>Mid: 0.91 (95% CI 0.87 - 0.96)<br>Late: 0.92 (95% CI 0.89 - 0.96)	NESI [5]	Pre: 0.80 (95% CrI 0.76 - 0.84)<br>Early: 0.84 (95% CrI 0.80 - 0.87)<br>Mid: 0.90 (95% CrI 0.87 - 0.91)<br>Late: 0.92 (95% CrI 0.90 - 0.94)
Proportion who have ever had an HIV test among PWID who have never been on OAT	Pre: 0.36 (95% CI 0.16 - 0.56)<br>Early: 0.38 (95% CI 0.21 - 0.55)<br>Mid: 0.41 (95% CI 0.24 - 0.59)<br>Late: 0.51 (95% CI 0.38 - 0.64)	NESI [5]	Pre: 0.31 (95% CrI 0.25 - 0.37)<br>Early: 0.33 (95% CrI 0.27 - 0.39)<br>Mid: 0.42 (95% CrI 0.37 - 0.48)<br>Late: 0.48 (95% CrI 0.42 - 0.54)
Proportion who have ever had an HIV test among PWID who have previously been on OAT but are not recently on OAT	Pre: 0.77 (95% CI 0.62 - 0.93)<br>Early: 0.83 (95% CI 0.64 - 1.00)<br>Mid: 0.93 (95% CI 0.84 - 1.00)<br>Late: 0.88 (95% CI 0.76 - 1.00)	NESI [5]	Pre: 0.87 (95% CrI 0.84 - 0.89)<br>Early: 0.89 (95% CrI 0.86 - 0.91)<br>Mid: 0.94 (95% CrI 0.92 - 0.95)<br>Late: 0.96 (95% CrI 0.95 - 0.97)

Table S2: Data used to calibrate the model. Outbreak eras are defined as: Pre = 2013-2014, Early = 2015-2016, Mid = 2017-2018, Late = 2019-2020. *(continued)*

Description	Value	Source	Posteriors
Proportion who have ever had an HIV test among PWID who are recently on OAT	Pre: 0.81 (95% CI 0.78 - 0.84)<br>Early: 0.89 (95% CI 0.86 - 0.91)<br>Mid: 0.92 (95% CI 0.90 - 0.95)<br>Late: 0.95 (95% CI 0.93 - 0.97)	NESI [5]	Pre: 0.85 (95% CrI 0.82 - 0.88)<br>Early: 0.88 (95% CrI 0.85 - 0.90)<br>Mid: 0.93 (95% CrI 0.91 - 0.94)<br>Late: 0.95 (95% CrI 0.94 - 0.96)
Number of HIV tests carried out in drug services in GGC by year.	2013: 746<br>2014: 912<br>2015: 1673<br>2016: 2616<br>2017: 2308<br>2018: 3610<br>2019: 4939	West of Scotland Specialist Virology Centre [4]	2013: 648 (95% CrI 422 - 905)<br>2014: 648 (95% CrI 422 - 907)<br>2015: 981 (95% CrI 740 - 1310)<br>2016: 1640 (95% CrI 1240 - 2250)<br>2017: 2070 (95% CrI 1550 - 2810)<br>2018: 2400 (95% CrI 1760 - 3270)<br>2019: 2740 (95% CrI 1960 - 3780)
Proportion of current injectors ever HIV tested in GCC.	Pre: 0.81 (95% CI 0.75 - 0.86)<br>Early: 0.84 (95% CI 0.79 - 0.90)<br>Mid: 0.94 (95% CI 0.90 - 0.98)<br>Late: 0.94 (95% CI 0.91 - 0.98)	NESI [5]	Pre: 0.84 (95% CrI 0.77 - 0.91)<br>Early: 0.88 (95% CrI 0.81 - 0.93)<br>Mid: 0.94 (95% CrI 0.90 - 0.96)<br>Late: 0.96 (95% CrI 0.94 - 0.97)
Proportion of current injectors ever HIV tested in RoGGC.	Pre: 0.79 (95% CI 0.75 - 0.82)<br>Early: 0.87 (95% CI 0.84 - 0.90)<br>Mid: 0.87 (95% CI 0.84 - 0.91)<br>Late: 0.90 (95% CI 0.87 - 0.92)	NESI [5]	Pre: 0.79 (95% CrI 0.76 - 0.82)<br>Early: 0.82 (95% CrI 0.78 - 0.85)<br>Mid: 0.87 (95% CrI 0.84 - 0.89)<br>Late: 0.90 (95% CrI 0.88 - 0.92)
Proportion of HIV diagnoses made in drugs services	2014: 0.22 (95% CI 0.03 - 0.54)<br>2015: 0.39 (95% CI 0.28 - 0.48)<br>2016: 0.39 (95% CI 0.26 - 0.52)<br>2017: 0.31 (95% CI 0.20 - 0.42)<br>2018: 0.41 (95% CI 0.23 - 0.58)<br>2019: 0.32 (95% CI 0.17 - 0.49)<br>(2020: 0.53 (95% CI 0.33 - 0.67))*<br>*Included to show general trend over time.	Estimate derived from proportion of HIV diagnoses in drugs services and prisons [1], and proportion of HIV tests carried out in drugs services vs. drugs services and prisons [4].	2014: 0.34 (95% CrI 0.21 - 0.47)<br>2015: 0.40 (95% CrI 0.29 - 0.48)<br>2016: 0.47 (95% CrI 0.39 - 0.52)<br>2017: 0.50 (95% CrI 0.45 - 0.55)<br>2018: 0.52 (95% CrI 0.47 - 0.56)<br>2019: 0.53 (95% CrI 0.49 - 0.57)
Number of HIV diagnoses	2014: 7<br>2015: 49<br>2016: 30<br>2017: 37<br>2018: 18<br>2019: 25	[1]	2014: 8.96 (95% CrI 6.15 - 13.4)<br>2015: 37.4 (95% CrI 25.4 - 53.5)<br>2016: 34.2 (95% CrI 25.1 - 45.8)<br>2017: 34.9 (95% CrI 25.0 - 47.4)<br>2018: 25.1 (95% CrI 16.6 - 36.3)<br>2019: 17.5 (95% CrI 10.4 - 28.0)

Table S2: Data used to calibrate the model. Outbreak eras are defined as: Pre = 2013-2014, Early = 2015-2016, Mid = 2017-2018, Late = 2019-2020. *(continued)*

Description	Value	Source	Posteriors
**Risk**			
Proportion of current injectors ever homeless in GCC.	0.90 (95% CI 0.78* - 0.98**)<br>*Min. 2.5th percentile across NESI surveys pre-late outbreak.<br>**Max. 97.5th percentile across NESI surveys pre-late outbreak.	NESI [5]	Pre: 0.89 (95% CrI 0.84 - 0.92)<br>Early: 0.89 (95% CrI 0.86 - 0.92)<br>Mid: 0.90 (95% CrI 0.86 - 0.93)<br>Late: 0.90 (95% CrI 0.86 - 0.94)
Proportion of current injectors ever homeless in RoGGC.	0.67 (95% CI 0.62* - 0.74**)<br>*Min. 2.5th percentile across NESI surveys pre-late outbreak.<br>**Max. 97.5th percentile across NESI surveys pre-late outbreak.	NESI [5]	Pre: 0.67 (95% CrI 0.64 - 0.72)<br>Early: 0.69 (95% CrI 0.64 - 0.76)<br>Mid: 0.71 (95% CrI 0.64 - 0.78)<br>Late: 0.72 (95% CrI 0.63 - 0.80)
Proportion of current injectors homeless in last 6 months GGC.	0.55 (95% CI 0.39* - 0.74**)<br>*Min. 2.5th percentile across NESI surveys pre-late outbreak.<br>**Max. 97.5th percentile across NESI surveys pre-late outbreak.	NESI [5]	Pre: 0.64 (95% CrI 0.52 - 0.73)<br>Early: 0.64 (95% CrI 0.52 - 0.74)<br>Mid: 0.65 (95% CrI 0.52 - 0.74)<br>Late: 0.65 (95% CrI 0.53 - 0.75)
Proportion of current injectors homeless in last 6 months in RoGGC.	0.19 (95% CI 0.11* - 0.27**)<br>*Min. 2.5th percentile across NESI surveys pre-late outbreak.<br>**Max. 97.5th percentile across NESI surveys pre-late outbreak.	NESI [5]	Pre: 0.18 (95% CrI 0.13 - 0.21)<br>Early: 0.18 (95% CrI 0.13 - 0.21)<br>Mid: 0.18 (95% CrI 0.13 - 0.22)<br>Late: 0.18 (95% CrI 0.13 - 0.22)
Proportion of current injectors injecting cocaine in GCC by outbreak period.	Pre: 0.29 (95% CI 0.23 - 0.35)<br>Early: 0.39 (95% CI 0.32 - 0.46)<br>Mid: 0.77 (95% CI 0.71 - 0.84)<br>Late: 0.77 (95% CI 0.70 - 0.84)	NESI [5]	Pre: 0.33 (95% CrI 0.25 - 0.41)<br>Early: 0.42 (95% CrI 0.31 - 0.51)<br>Mid: 0.70 (95% CrI 0.53 - 0.81)<br>Late: 0.77 (95% CrI 0.67 - 0.84)
Proportion of current injectors injecting cocaine in RoGGC by outbreak period.	Pre: 0.21 (95% CI 0.17 - 0.24)<br>Early: 0.21 (95% CI 0.18 - 0.24)<br>Mid: 0.40 (95% CI 0.35 - 0.45)<br>Late: 0.51 (95% CI 0.47 - 0.55)	NESI [5]	Pre: 0.19 (95% CrI 0.16 - 0.23)<br>Early: 0.20 (95% CrI 0.16 - 0.24)<br>Mid: 0.32 (95% CrI 0.25 - 0.39)<br>Late: 0.52 (95% CrI 0.38 - 0.63)
**OAT**			
Proportion of current injectors who have ever been on OAT.	0.91 (95% CI 0.84* - 0.97**)<br>*Min. 2.5th percentile across NESI surveys pre-late outbreak.<br>**Max. 97.5th percentile across NESI surveys pre-late outbreak.	NESI [5].	Pre: 0.91 (95% CrI 0.89 - 0.93)<br>Early: 0.91 (95% CrI 0.89 - 0.93)<br>Mid: 0.91 (95% CrI 0.89 - 0.93)<br>Late: 0.92 (95% CrI 0.90 - 0.94)

Table S2: Data used to calibrate the model. Outbreak eras are defined as: Pre = 2013-2014, Early = 2015-2016, Mid = 2017-2018, Late = 2019-2020. *(continued)*

Description	Value	Source	Posteriors
Proportion of current injectors on OAT in the last 6 months.	0.84 (95% CI 0.77* - 0.92**)<br>*Min. 2.5th percentile across NESI surveys pre-late outbreak.<br>**Max. 97.5th percentile across NESI surveys pre-late outbreak.	NESI [5].	Pre: 0.83 (95% CrI 0.79 - 0.87)<br>Early: 0.84 (95% CrI 0.79 - 0.87)<br>Mid: 0.84 (95% CrI 0.80 - 0.88)<br>Late: 0.84 (95% CrI 0.80 - 0.88)

4 Model fit

The model appears to fit the calibration data well, as illustrated by the Figures S2 and S3.

Figure S2 shows that the increase in HIV prevalence among different risk groups is captured, with higher HIV prevalence among people injecting cocaine, people who are homeless and people in GCC. The impact of OAT on HIV wasn't calibrated to the OAT prevalence data; an informed prior distribution was used instead. This is appropriate for the Glasgow HIV outbreak, where most people are on OAT.

Figure S3 shows model predictions relating to HIV testing and risk categories compared to relevant data. The increase in systematic testing is captured, with appropriately wide credibility intervals, particularly for later in the outbreak. The cumulative number of diagnoses over the course of the outbreak matches the data well. The increase in cocaine injecting, which differs between GCC and RoGGC, has been captured, albeit with wide credibility intervals. Sensitivity analysis on these parameters suggests they make little difference to the scenario analysis results, as demonstrated in Section 5. The proportions of people who are recently/have ever been homeless in GCC and RoGGC also fit well, with substantial overlap between the model credibility intervals and the confidence intervals for the data.

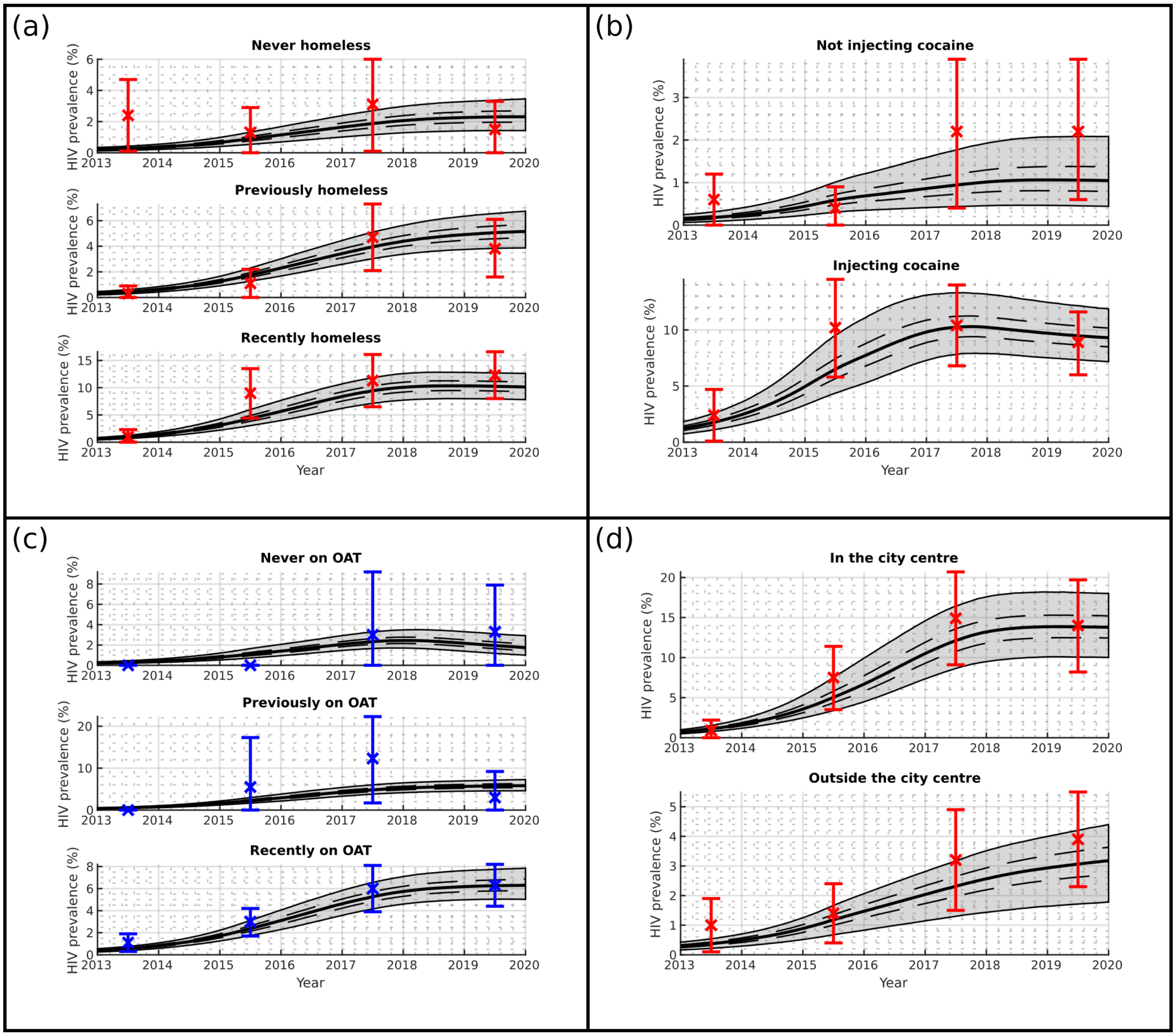

Figure S2: Example illustrations of HIV prevalence model fits; (a) by homelessness status, (b) by cocaine injecting status, (c) by OAT status, and (d) by geography status. Red data points were calibrated to, blue data points were not.

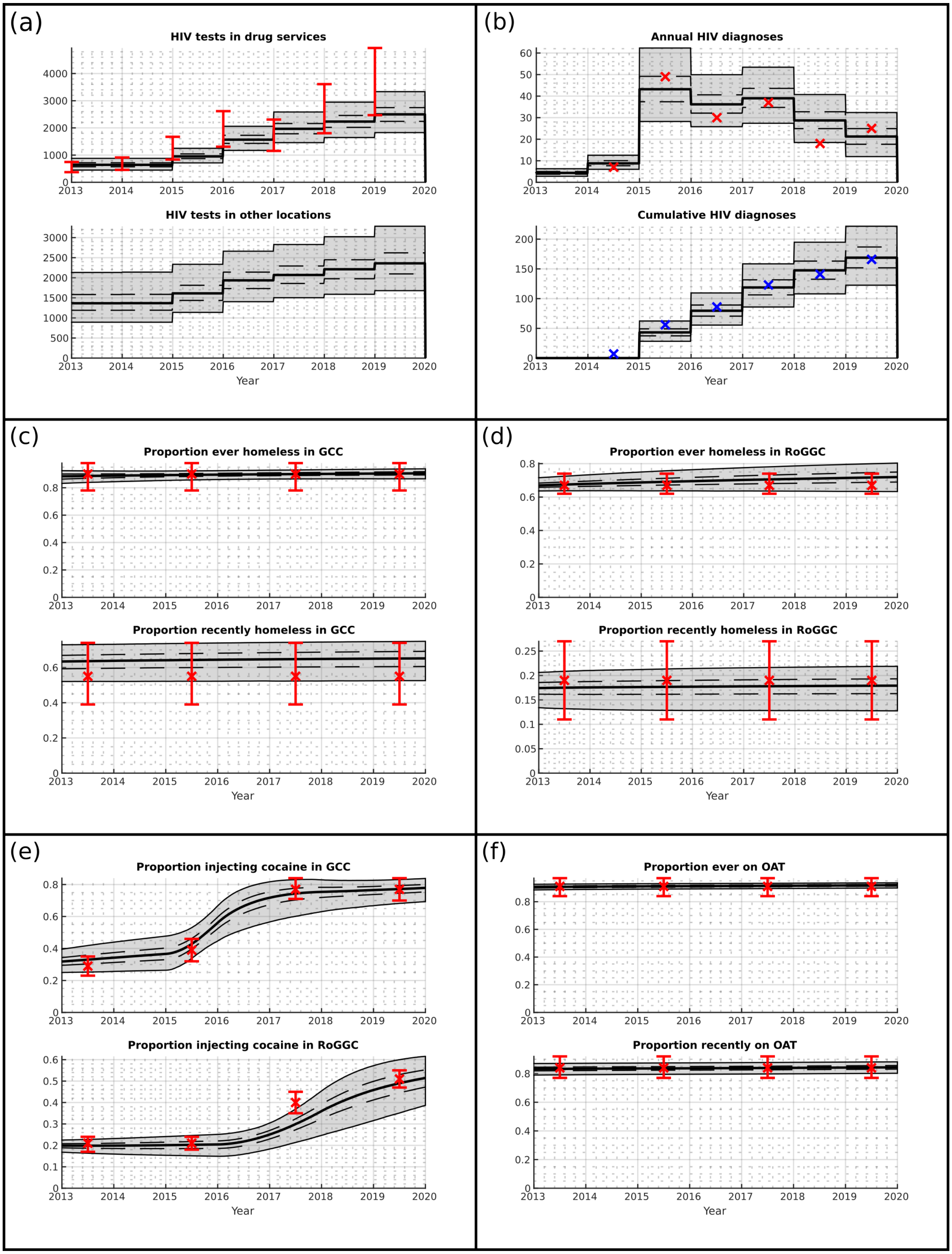

Figure S3: More example illustrations of model fits; (a) HIV tests, (b) HIV diagnoses, (c) proportions homeless in GCC, (d) proportions homeless in RoGGC, (e) proportions injecting cocaine by geography status, and (f) proportions on OAT. Red data points were calibrated to, blue data points were not.

5 Sensitivity Analysis

5.1 Methods

We carry out one-way sensitivity analysis on the model results for a selection of key parameters relating to HIV transmission, HIV testing, cocaine injecting and geography. First, we draw a random sample of 10% of the calibrated parameter sets. Then, for each parameter set, we vary each key parameter in turn within the range of its prior distribution (or a specified range if the parameter was fixed in the model), calculating outputs for each scenario. For each value of the varying parameter, we take the median of the model outputs across the parameter sets. Then, we calculate the minimum, median and maximum of these values across the full range of the varying parameter. The results are reported as forest plots, alongside the variation across the calibrated data sets for comparison. This approach demonstrates how sensitive the estimated impact of the interventions is to variation in the key parameters.

Note that the sensitivity analysis is not an assessment of uncertainty. The calibration algorithm finds posterior distributions for the parameter values which fit the data. The model credibility intervals provide an assessment of uncertainty based on these calibrated parameter values. The sensitivity analysis can be used to understand which of these posterior distributions is a priority for validation.

We are using the prior distribution ranges to carry out the sensitivity analysis. If the model is particularly sensitive to one parameter, it is likely to be given a narrower posterior distribution, which makes the posterior less suitable for a sensitivity investigation. Prior distributions for parameters not informed by data have large ranges, and are therefore more likely to contain values which are not representative of the real-world. More data is required to validate the posterior distributions for parameters where wide prior distributions lead to large variation in the model output.

5.2 Findings

Figures S4 and S5 illustrate forest plots showing how variation in key parameters affects the increase in prevalence at the beginning of 2020 between the GECHO+ scenario and each of the comparison scenarios. Figures S6 and S7 illustrate how the parameters affect the corresponding increase in incidence for each comparison scenario, and figures S8 and S9 show how the number of cases averted is affected.

Variation in parameters related to HIV transmission, such as for cocaine injecting assortative mixing, the homeless effect on HIV, the cocaine effect on HIV and the per capita infection rate, consistently leads to a large range of output values across the different output measures. Furthermore, the range of outputs for these parameter values often falls outside of the range of variation in the calibrated output. This suggests a high degree of variability in the GECHO+ scenario due to changes in these parameter values, i.e. the parameter range leads to model outputs which fit poorly to the calibration data, or strong co-dependency between parameter values.

As expected, variation in the population size leads to a large range of outputs values for the number of cases averted.

Demographic parameters, such as those related to geography and cocaine injecting, appear to have little impact on the output measures investigated.

5.3 Discussion

We would expect the model to be sensitive to parameters relating to the HIV transmission rate, as these parameters drive the outbreak. However, these parameters generally have wide prior distributions which is contributing to the observed impact on model outputs. Moreover, there are several parameters related to HIV transmission, particularly for different risk groups. More data to inform smaller ranges on the prior distributions for these parameters would help reduce the observed variation in model output. For example, we have assumed that OAT affects HIV transmission for PWID injecting cocaine in the same way as for PWID not injecting cocaine, but that PWID injecting cocaine are not more likely to cease injecting if they are on OAT. This may mean that the model calibration algorithm is overestimating the cocaine effect on

HIV transmission to compensate. As the sensitivity analysis suggests that the model output is sensitive to this parameter, it is particularly important for data related to cocaine injecting and HIV transmission to be produced to better understand this assumption. Additionally, the posterior parameter distribution suggests a high degree of assortative mixing by cocaine injecting; it is skewed towards the higher values compared to the prior distribution. The sensitivity analysis suggests the results are sensitive to changes in this parameter. This provides further evidence that additional data related to cocaine injecting and HIV transmission is required to validate the results of the calibration algorithm.

More detailed data on the modelled population size would allow a more accurate prediction of the number of cases averted. Uncertainty in the population size highlights the importance of presenting relative results were possible, hence our focus on the percentage change in prevalence/incidence due to the interventions.

A main drawback of the one-way sensitivity analysis approach taken is that doesn't look at the impact of all of the parameters. Due to the large number of parameters in the model, we considered it to be appropriate to focus on the parameters more directly related to HIV transmission/acquisition and the interventions being analysed in this study. Furthermore, the sensitivity analysis approach doesn't account for relationships between the parameters, e.g. if the cocaine effect on transmission is calibrated to a higher value then the proportion of assortative mixing by cocaine injecting is likely to take a lower value. It would be appropriate to carry out some structural sensitivity analysis to investigate how sensitive the results are to changes in model structure. The sensitivity analysis also doesn't account for the model fit of the GECHO+ scenario to the calibration data. For this reason, we remain confident in our results despite some highly sensitive parameters; the calibration process accounts for relationships between parameters and narrows parameter ranges appropriately to fit the calibration data. However, we emphasise that the highly sensitive parameters are a priority for validation using data which may become available in the future.

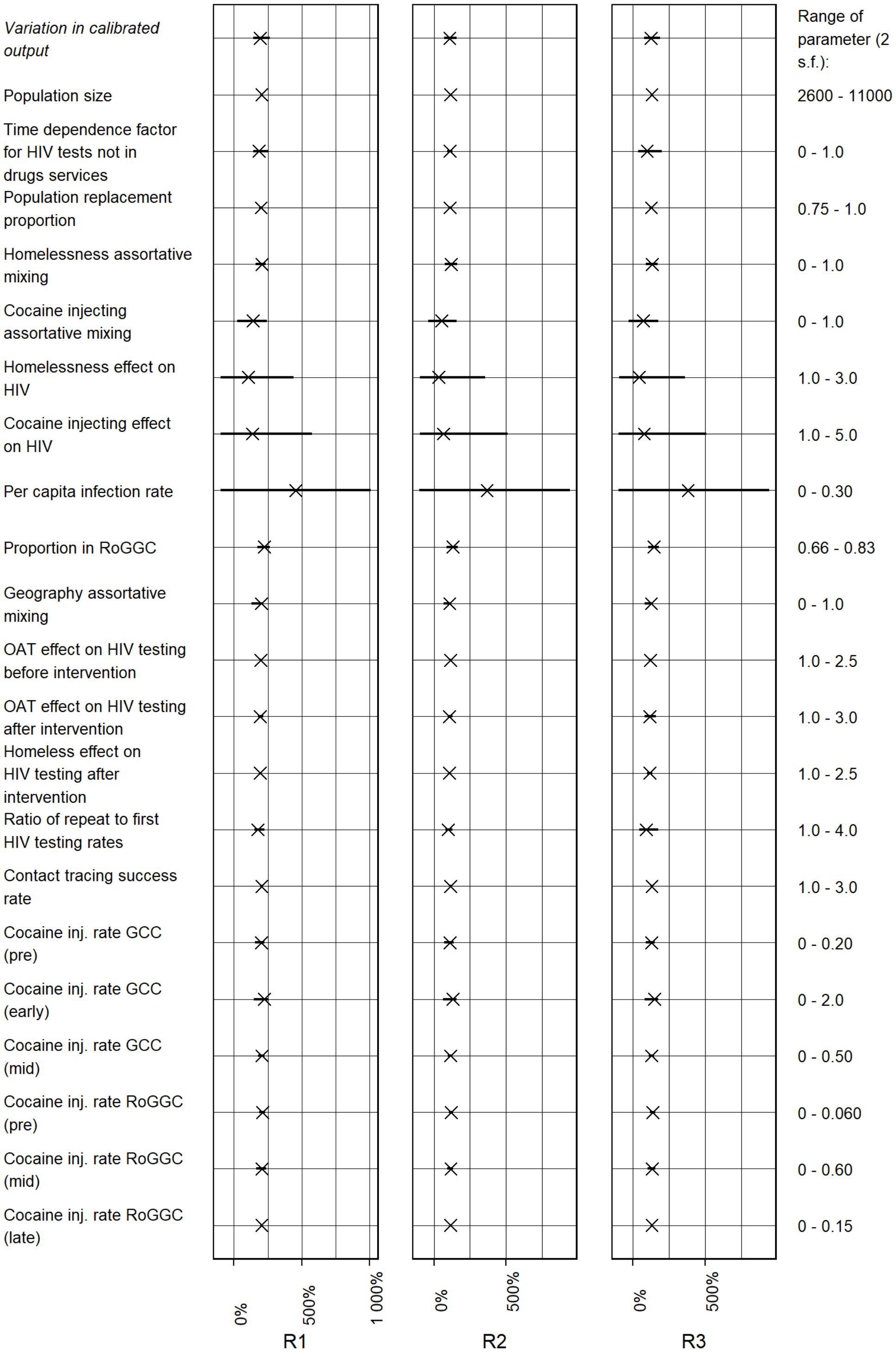

Figure S4: Forest plots illustrating how the increase in prevalence at the beginning of 2020 between the baseline and comparison scenarios (R1 - Removing HIV testing and treatment improvements, R2 = Removing improvements in HIV treatment, R3 = Removing improvements in HIV testing) changes as individual parameters are varied (labelled on the left). A value of 0% indicates that the baseline and comparison scenarios predict the same prevalence at the beginning of 2020. For comparison, the variation due to uncertainty in the calibrated parameter values is also illustrated.

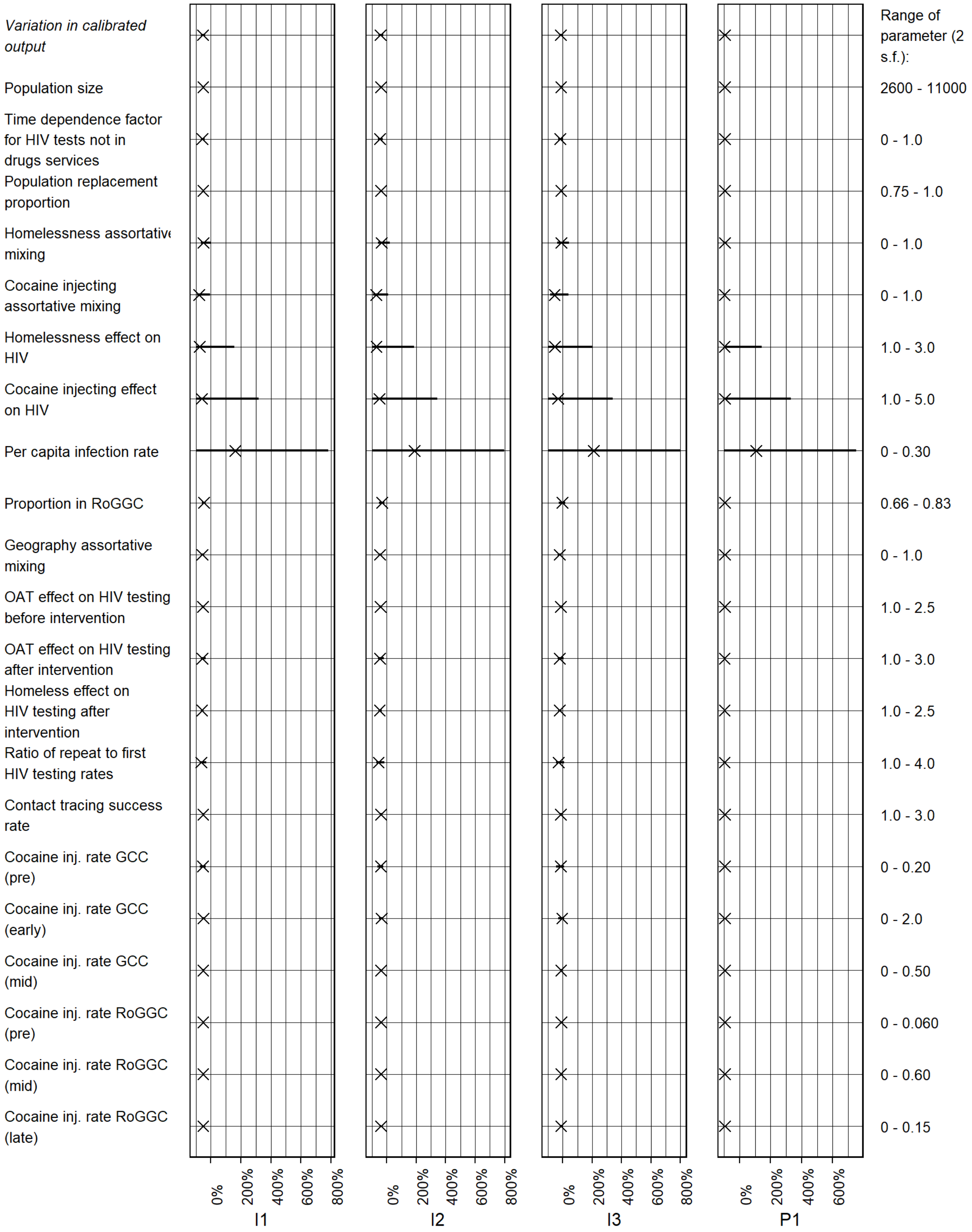

Figure S5: Forest plots illustrating how the increase in prevalence at the beginning of 2020 between the baseline and comparison scenarios (I1 - Immediate improvements in HIV testing and treatment interventions, I2 = Immediate improvement in HIV treatment, I3 = Immediate improvement in HIV testing, P1 - Pre-emptive improvements in HIV testing and treatment interventions) changes as individual parameters are varied (labelled on the left). A value of 0% indicates that the baseline and comparison scenarios predict the same prevalence at the beginning of 2020. For comparison, the variation due to uncertainty in the calibrated parameter values is also illustrated.

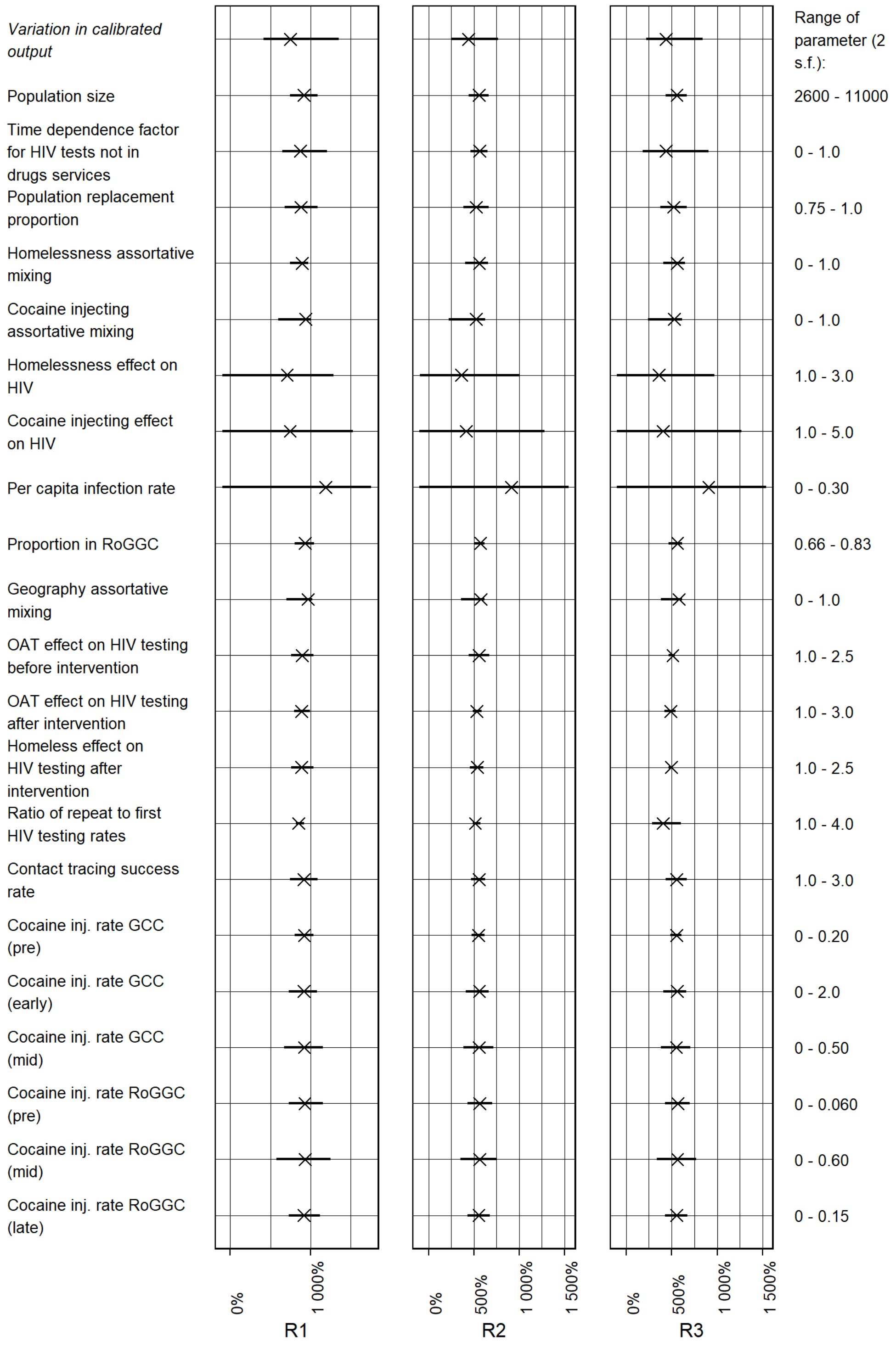

Figure S6: Forest plots illustrating how the increase in incidence at the beginning of 2020 between the baseline and comparison scenarios (R1 - Removing HIV testing and treatment improvements, R2 = Removing improvements in HIV treatment, R3 = Removing improvements in HIV testing) changes as individual parameters are varied (labelled on the left). A value of 0% indicates that the baseline and comparison scenarios predict the same incidence at the beginning of 2020. For comparison, the variation due to uncertainty in the calibrated parameter values is also illustrated.

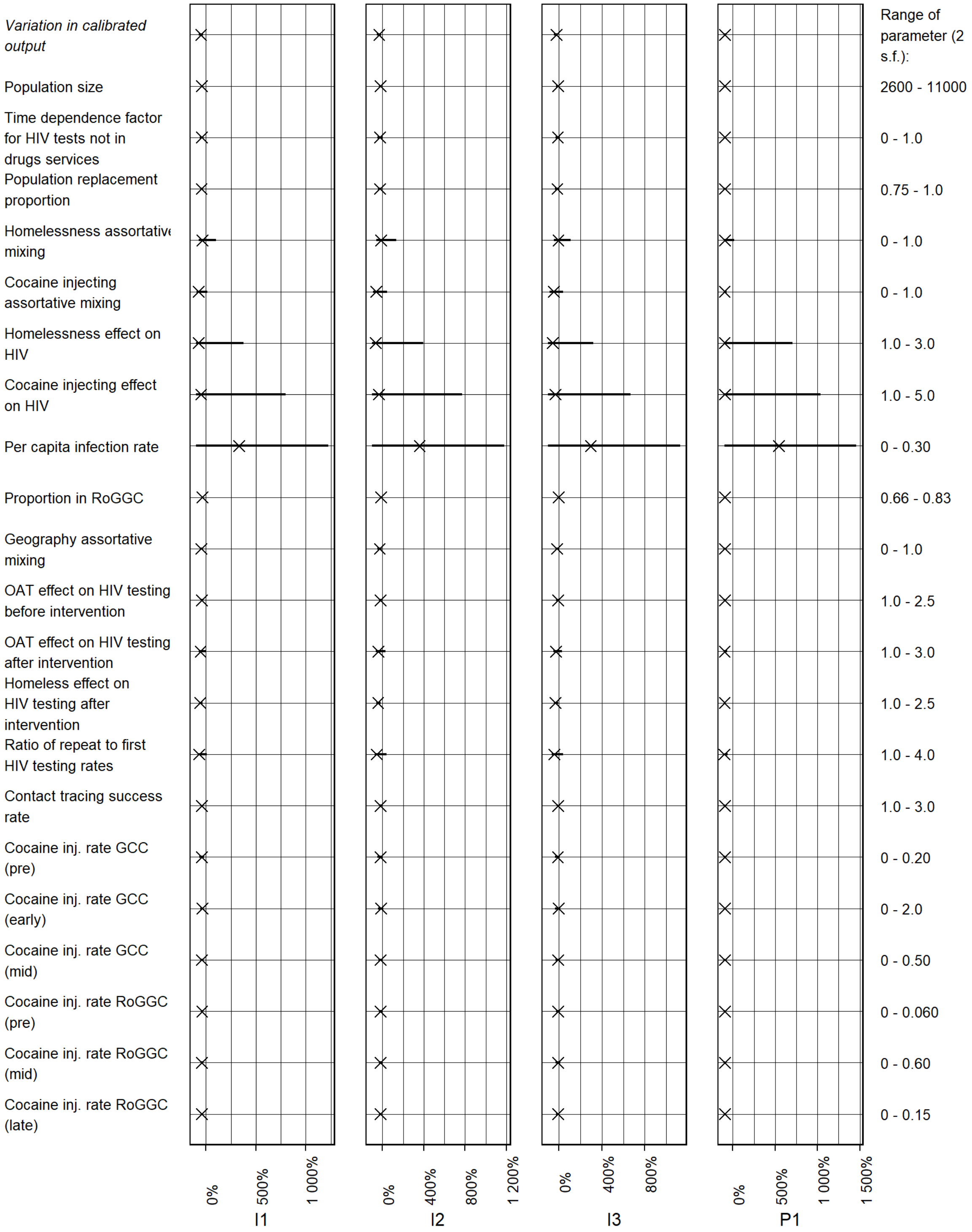

Figure S7: Forest plots illustrating how the increase in incidence at the beginning of 2020 between the baseline and comparison scenarios (I1 - Immediate improvements in HIV testing and treatment interventions, I2 = Immediate improvement in HIV treatment, I3 = Immediate improvement in HIV testing, P1 - Pre-emptive improvements in HIV testing and treatment interventions) changes as individual parameters are varied (labelled on the left). A value of 0% indicates that the baseline and comparison scenarios predict the same incidence at the beginning of 2020. For comparison, the variation due to uncertainty in the calibrated parameter values is also illustrated.

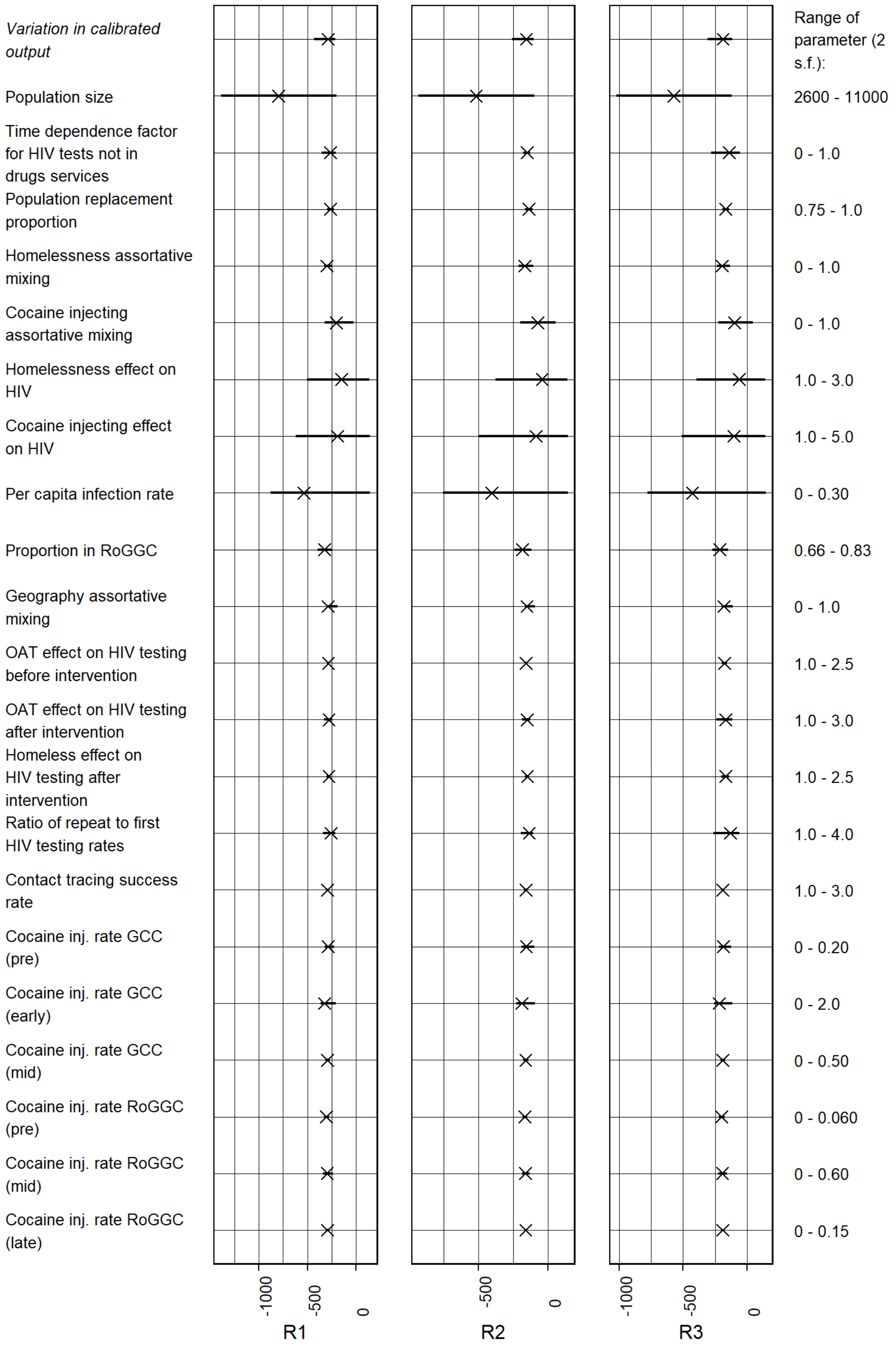

Figure S8: Forest plots illustrating how the cases averted for different counterfactual scenarios (R1 - Removing HIV testing and treatment improvements, R2 = Removing improvements in HIV treatment, R3 = Removing improvements in HIV testing) changes as individual parameters are varied (labelled on the left). For comparison, the variation due to uncertainty in the calibrated parameter values is also illustrated.

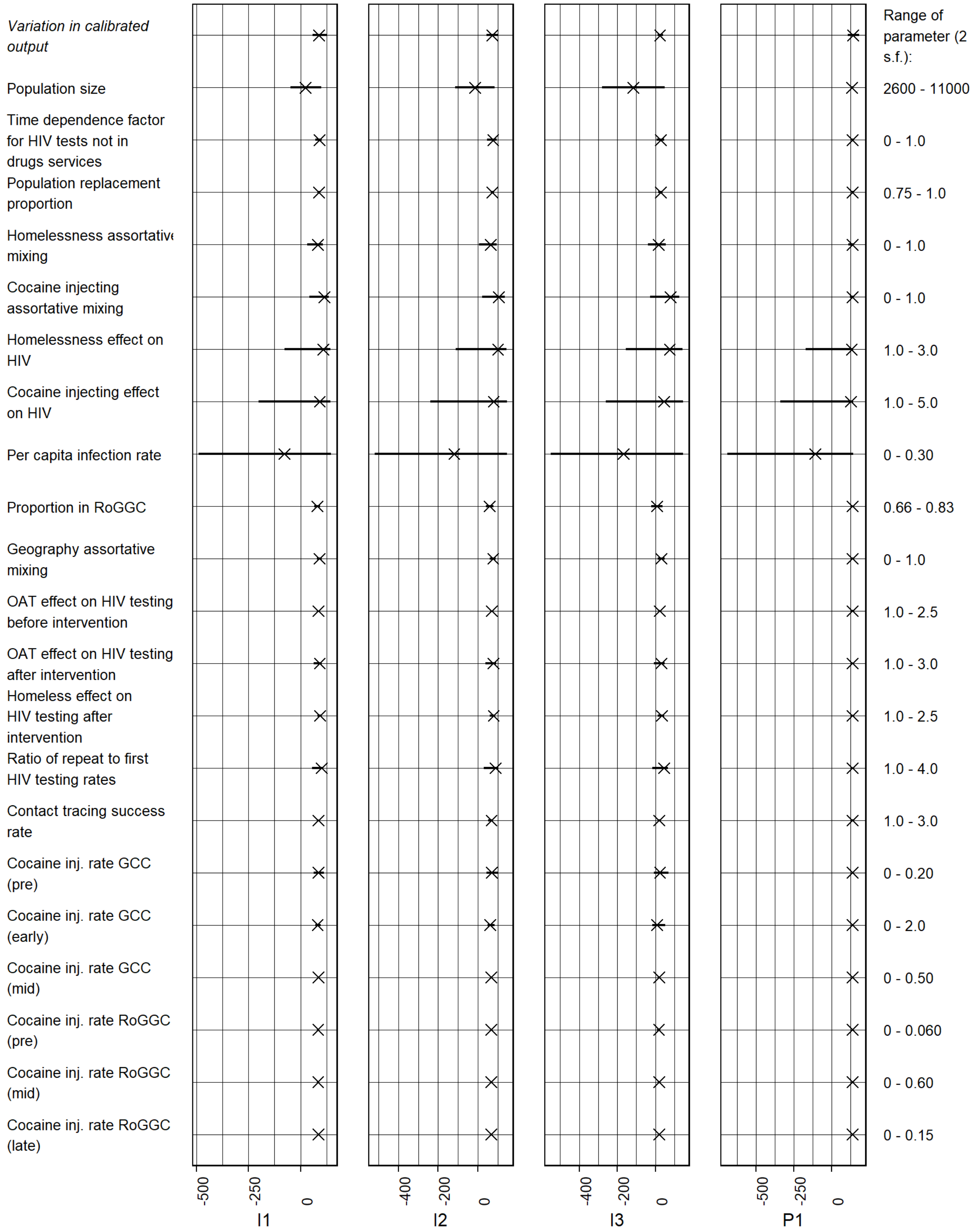

Figure S9: Forest plots illustrating how the cases averted for different counterfactual scenarios (I1 - Immediate improvements in HIV testing and treatment interventions, I2 = Immediate improvement in HIV treatment, I3 = Immediate improvement in HIV testing, P1 - Pre-emptive improvements in HIV testing and treatment interventions) changes as individual parameters are varied (labelled on the left). For comparison, the variation due to uncertainty in the calibrated parameter values is also illustrated.

References

[1] *Linked data from the National HIV diagnosis database at PHS with the NHS GGC Laboratory HIV test data from 2000 – Aug 2020.*

[2] *Time from HIV-1 seroconversion to AIDS and death before widespread use of highly-active antiretroviral therapy: a collaborative re-analysis. Collaborative Group on AIDS Incubation and HIV Survival including the CASCADE EU Concerted Action.*, Lancet, 355 (2000), pp. 1131–7.

[3] *ART and viral suppression data for NHS Greater Glasgow and Clyde*, 2020.

[4] *HIV testing data for Greater Glasgow and Clyde*, 2020.

[5] *Public Health Scotland, Glasgow Caledonian University and the West of Scotland Specialist Virology Centre. The Needle Exchange Surveillance Initiative (NESI): Prevalence of blood-borne viruses and injecting risk behaviours among people who inject drugs attending injecting equipment provision services in Scotland, 2008 to 2020*, 2022.

[6] C. C. Drovandi and A. N. Pettitt, *Estimation of parameters for macroparasite population evolution using approximate Bayesian computation*, Biometrics, 67 (2011), pp. 225–33.

[7] E. Flountzi, A. G. Lim, P. Vickerman, D. Paraskevis, M. Psichogiou, A. Hatzakis, and V. Sypsa, *Modeling the impact of interventions during an outbreak of HIV infection among people who inject drugs in 2012–2013 in Athens, Greece*, Drug and Alcohol Dependence, 234 (2022).

[8] L. Gao, J. R. Robertson, and S. M. Bird, *Non drug-related and opioid-specific causes of 3262 deaths in Scotland's methadone-prescription clients, 2009-2015*, Drug Alcohol Depend, 197 (2019), pp. 262–270.

[9] G. Hay, M. Gannon, J. Casey, and N. McKeganey, *Estimating the National and Local Prevalence of Problem Drug Misuse in Scotland*, report, University of Glasgow, 2009.

[10] T. D. Hollingsworth, R. M. Anderson, and C. Fraser, *HIV-1 transmission, by stage of infection*, J Infect Dis, 198 (2008), pp. 687–93.

[11] P. A. Kemp, J. Neale, and M. Robertson, *Homelessness among problem drug users: prevalence, risk factors and trigger events*, Health Soc Care Community, 14 (2006), pp. 319–28.

[12] J. Kimber, L. Copeland, M. Hickman, J. Macleod, J. McKenzie, D. De Angelis, and J. R. Robertson, *Survival and cessation in injecting drug users: prospective observational study of outcomes and effect of opiate substitution treatment*, BMJ, 341 (2010), p. c3172.

[13] G. J. MacArthur, S. Minozzi, N. Martin, P. Vickerman, S. Deren, J. Bruneau, L. Degenhardt, and M. Hickman, *Opiate substitution treatment and HIV transmission in people who inject drugs: systematic review and meta-analysis*, BMJ, 345 (2012), p. e5945.

[14] M. T. May, S. M. Ingle, D. Costagliola, A. C. Justice, F. de Wolf, M. Cavassini, A. D'Arminio Monforte, J. Casabona, R. S. Hogg, A. Mocroft, F. C. Lampe, F. Dabis, G. Fätkenheuer, T. R. Sterling, J. del Amo, M. J. Gill, H. M. Crane, M. S. Saag, J. Guest, H. R. Brodt, and J. A. Sterne, *Cohort profile: Antiretroviral Therapy Cohort Collaboration (ART-CC)*, Int J Epidemiol, 43 (2014), pp. 691–702.

[15] A. McAuley, N. E. Palmateer, D. J. Goldberg, K. M. A. Trayner, S. J. Shepherd, R. N. Gunson, R. Metcalfe, C. Milosevic, A. Taylor, A. Munro, and S. J. Hutchinson, *Re-emergence of HIV related to injecting drug use despite a comprehensive harm reduction environment: a cross-sectional analysis*, The Lancet HIV, 6 (2019), pp. e315–e324.

[16] S. A. McDonald, A. McAuley, M. Hickman, S. M. Bird, A. Weir, K. Templeton, R. Gunson, and S. J. Hutchinson, *Increasing drug-related mortality rates over the last decade in Scotland are not just due to an ageing cohort: A retrospective longitudinal cohort study*, Int J Drug Policy, 96 (2021), p. 103286.

[17] R. Metcalfe, M. Ragonnet-Cronin, A. Bradley-Stewart, A. McAuley, H. Stubbs, T. Ritchie, R. O'Hara, K. Trayner, C. Glover, L. Laverty, L. Sills, K. Brown, R. Gunson, J. Campbell, C. Milsoevic, P. Anderson, and S. E. Peters, *From Hospital to the Community: Redesigning the Human Immunodeficiency Virus (HIV) Clinical Service Model to Respond to an Outbreak of HIV Among People Who Inject Drugs*, J Infect Dis, 222 (2020), pp. S410–S419.

[18] B. Nosyk, J. E. Min, E. Evans, L. Li, L. Liu, V. D. Lima, E. Wood, and J. S. Montaner, *The Effects of Opioid Substitution Treatment and Highly Active Antiretroviral Therapy on the Cause-Specific Risk of Mortality Among HIV-Positive People Who Inject Drugs*, Clin Infect Dis, 61 (2015), pp. 1157–65.

[19] T. C. Quinn, M. J. Wawer, N. Sewankambo, D. Serwadda, C. Li, F. Wabwire-Mangen, M. O. Meehan, T. Lutalo, and R. H. Gray, *Viral load and heterosexual transmission of human immunodeficiency virus type 1. Rakai Project Study Group*, N Engl J Med, 342 (2000), pp. 921–9.

[20] M. Ragonnet-Cronin, C. Jackson, A. Bradley-Stewart, C. Aitken, A. McAuley, N. Palmateer, R. Gunson, D. Goldberg, C. Milosevic, and A. J. Leigh Brown, *Recent and Rapid Transmission of HIV Among People Who Inject Drugs in Scotland Revealed Through Phylogenetic Analysis*, J Infect Dis, 217 (2018), pp. 1875–1882.

[21] S. Sturniolo, W. Waites, T. Colbourn, D. Manheim, and J. Panovska-Griffiths, *Testing, tracing and isolation in compartmental models*, PLoS Comput Biol, 17 (2021), p. e1008633.

[22] T. Toni, D. Welch, N. Strelkowa, A. Ipsen, and M. P. Stumpf, *Approximate Bayesian computation scheme for parameter inference and model selection in dynamical systems*, J R Soc Interface, 6 (2009), pp. 187–202.

[23] K. M. A. Trayner, N. E. Palmateer, A. McAuley, R. Metcalfe, D. J. Goldberg, E. Peters, J. Craik, R. N. Gunson, D. Thain, D. Carter, and S. J. Hutchinson, *Evaluation of the scale-up of HIV testing among people who inject drugs in Scotland in the context of an ongoing HIV outbreak*, Int J Drug Policy, (2021), p. 103304.

[24] Y. Xia, S. Seaman, M. Hickman, J. Macleod, R. Robertson, L. Copeland, J. McKenzie, and D. De Angelis, *Factors affecting repeated cessations of injecting drug use and relapses during the entire injecting career among the Edinburgh Addiction Cohort*, Drug Alcohol Depend, 151 (2015), pp. 76–83.
